# Identification of Mouse Serum miRNA Endogenous References by Global Gene Expression Profiles

**DOI:** 10.1371/journal.pone.0031278

**Published:** 2012-02-10

**Authors:** Qing-Sheng Mi, Matthew Weiland, Rui-Qun Qi, Xing-Hua Gao, Laila M. Poisson, Li Zhou

**Affiliations:** 1 Henry Ford Immunology Program, Henry Ford Health System, Detroit, Michigan, United States of America; 2 Department of Dermatology, Henry Ford Health System, Detroit, Michigan, United States of America; 3 Department of Internal Medicine, Henry Ford Health System, Detroit, Michigan, United States of America; 4 Department of Dermatology, No 1 Hospital, China Medical University, Shenyang, China; 5 Department of Public Health Sciences, Henry Ford Health System, Detroit, Michigan, United States of America; Radboud University, Netherlands

## Abstract

MicroRNAs (miRNAs) are recently discovered small non-coding RNAs and can serve as serum biomarkers for disease diagnosis and prognoses. Lack of reliable serum miRNA endogenous references for normalization in miRNA gene expression makes single miRNA assays inaccurate. Using TaqMan® real-time PCR miRNA arrays with a global gene expression normalization strategy, we have analyzed serum miRNA expression profiles of 20 female mice of NOD/ShiLtJ (n = 8), NOR/LtJ (n = 6), and C57BL/6J (n = 6) at different ages and disease conditions. We identified five miRNAs, miR-146a, miR-16, miR-195, miR-30e and miR-744, to be stably expressed in all strains, which could serve as mouse serum miRNA endogenous references for single assay experiments.

## Introduction

MicroRNAs (miRNAs) are a recently discovered class of 21–25 nt single stranded non-coding RNAs, which are widely expressed in plants, animals, and humans, and function through translational repression of specific target mRNAs. As regulators of gene expression, miRNAs act in a complementary fashion by partially binding to the 3′ untranslated region of target mRNAs [1]. Repression of protein translation follows the pairing of miRNAs to target sequences. The unique mechanism of gene regulation make miRNAs an important regulator of a diverse set of physiological processes [2], [3], including immune cell development [3], [4], [5]. miRNAs have been found to be dysregulated in multiple disease states, most notably in cancer where expression of miRNAs appear to be tissue-specific [6], [7].

Circulating nucleic acids were first described by Mandel and Metais in 1948, when nucleic acids were measured in human plasma [8]. The potential use of circulating nucleic acids extended to disease diagnosis much later, when increased levels of serum DNA distinguished cancer patients from healthy controls [9]. In addition, the possibility of nucleic acids as evaluators of therapeutic effectiveness was also reported, levels of serum DNA in cancer patients decreased when treatment was effective [10], [11]. Recent studies have demonstrated that serum and plasma also contain a large amount of stable miRNAs and have potential to serve as biomarkers for changes in physiological and pathological conditions, such as pregnancy, cancer, and other diseases [12], [13], [14], [15], [16]. Circulating miRNAs show remarkable stability and demonstrate consistent expression profiles between healthy controls and patients [12], [13], [15], [16]. The extraction and quantification of serum miRNAs is a challenging task, however miRNA detection is much easier than the detection of some current protein biomarkers. Although most studies have focused on using circulating miRNAs as biomarkers in human, circulating miRNAs in rodent models are able to distinguish healthy animals from diseased animals as well [14], [17], [18], [19].

The exciting research of circulating miRNA biomarkers has a great potential; however, the issue currently at the forefront of serum miRNA research is proper normalization of miRNA gene expression with invariant housekeeping genes. Normalization of circulating miRNA remains a significant hurdle that must be addressed in order to facilitate biomarker discovery and fully validate single miRNA quantitative real-time PCR (qRT-PCR) assays. It is important to ensure that gene expression differences are a direct result of the diseases under study and not due to other sources of variation [20]. Uncontrolled variation can be limited through normalization. Equal amounts of total RNA are commonly used as the initial normalizer when conducting qRT-PCR experiments. However, the total amount of miRNA extracted from even the same quantities of serum or plasma from different samples is generally not equal. Research has been done to discover normalizers of miRNA expression in tissue samples, which addresses the variation of RNA loading amounts between samples. For example Peltier and Latham reported expression of tissue miR-191 and miR-103 to be more consistent than commonly used small RNAs and even total RNA [20]. Even though miRNA normalizers may be present in tissue, endogenous references specific to circulating miRNAs have not been identified. In this report, we have evaluated the serum miRNA profiles of 20 individual mice from different strains using a TaqMan® Array MicroRNA real-time PCR strategy in order to identify the miRNAs with the most stable expression in the serum of mouse. These samples represent multiple strains of mice at different ages as well as different conditions, including autoimmune diabetes. Microarray analysis of 277 miRNAs, using global normalization and subsequent data filters, identified five miRNAs as circulating miRNA endogenous references.

## Methods

### Mouse

Female NOD/ShiLtJ (NOD n = 8, stock# 001976), NOR/LtJ (NOR n = 6, stock# 002050), and C57BL/6J (B6 n = 6, stock# 000664) (Jackson Laboratory, Bar Harbor, Maine) mice were used for all experiments. NOD mice spontaneously develop T-cell mediated insulin-dependent diabetes mellitus staring at 14–15 weeks old, while NOR mice only develop pancreatic insulitis without diabetes development. All animal studies have been approved by Institutional Animal Care and Use Committee (IACUC).

### Serum Collection

Mouse blood was collected following euthanization via heart puncture. Blood was allowed to clot for at least 1 hour, centrifuged for 10 minutes×3,000 rpm, and supernatant (serum) was removed. Centrifugation was repeated for 10 minutes×2,500 rpm and the resulting serum layer was used for RNA isolation or stored at −80°C until further use. For NOD mice, serum was collected at 3–4 weeks, 7–8 weeks, 16–19 weeks (non-diabetic), and after developing diabetes. For NOR and B6 mice, serum were collected at 3–4 weeks, 7–8 weeks, and 16–19 weeks. Each time point from each mouse strain was duplicated.

### RNA Extraction and TaqMan® Low-Density Array miRNA qRT-PCR

RNA was isolated from serum samples by using a combination of Trizol® LS (Invitrogen™, Carlsbad, CA) and the mirVana™ miRNA Isolation kit (Ambion®, Austin, TX) or the Qiagen® miRNeasy® Mini kit (Qiagen Inc.®, Germantown, MD) with some modifications. Each sample was measured by a NanoDrop 2000 spectrophotometer (Thermo Scientific). The RNA (about 50 ng) was reverse transcribed using the TaqMan® MiRNA Reverse Transcription Kit (Applied Biosystems, Foster City, CA, USA), and the TaqMan® MiRNA Multiplex RT Assays, Rodent pool A (v2.0) (Applied Biosystems). 3 µl of RNA was added to each reaction and RT-PCR was carried out on the ABI Veriti™ Thermal cycler (Applied Biosystems). 2.5 µl of the product from each reverse transcription reaction was preamplified per the manufacturer's protocol with the Megaplex™ PreAmp Primers (10×), Rodent pool A (v2.0), and TaqMan® PreAmp Master Mix (2×) using the ABI Veriti™ (Applied Biosystems). Following preamplification the miRNA expression was profiled with TaqMan® Rodent MicroRNA array card A v2.0, performed on a 7900HT Fast Real-Time PCR System (Applied Biosystems), using the manufacturer's recommended protocol.

### Global Normalization and Statistical Analysis

Raw cycle threshold (C_T_) values were calculated using SDS 2.3 and RQ manager 1.2 software (Applied Biosystems) applying automatic baselines and threshold settings. The C_T_ values were imported into StatMiner® 4.2 (Integromics® Inc., Philadelphia, PA) for global normalization of each sample. The miRNAs that were detected in all 20 samples were selected for the reference set in global normalization [21]. Within each sample, the global normalization process subtracts the mean C_T_ value of the reference set from the C_T_ value of each miRNA from the same sample. The resulting value is the ΔC_T_. Endogenous controls must be consistently expressed, thus only the miRNAs that were expressed in all samples were considered to be candidates and subjected to further data analysis. These ΔC_T_ values were analyzed with one-way analysis of variance (ANOVA) using mouse strain as the predictor and the Least-significant difference method for pair-wise comparisons using SPSS 18.0.0 (SPSS Inc., Chicago, IL). The miRNAs with ANOVA *p*-values greater than 0.3, demonstrating no significant difference between strains, were retained as endogenous reference candidates. Endogenous references should have a small variation among all samples. Therefore the standard deviation (SD) across all samples was calculated using SPSS 18.0.0. miRNAs with an SD less than 1 were selected. Finally, in order to increase the strictness of our criteria the pair-wise |ΔΔC_T_| of the candidate miRNAs were analyzed to determine the difference in mean ΔC_T_ values within the 3 strains. Candidate miRNAs had |ΔΔC_T_| values less than 0.5. Consequently genes with an ANOVA *p*>0.3, an SD<1, and all pair-wise |ΔΔC_T_|<0.5 were selected as endogenous reference genes.

## Results

### Identification of serum miRNA endogenous references

Our data is based on 20 serum miRNA expression profiles from B6, autoimmune-prone NOD, and NOR mouse strains, across different ages, and with diabetes and pancreatic inflammation. A total of 277 miRNAs were expressed between all serum samples in the TaqMan® Array MicroRNA Rodent A card, which contains 335 potential target miRNAs. The ΔC_T_ values for each miRNA were calculated using the global normalization option of the StatMiner® software. The global normalization method is convenient for high-throughput miRNA assays where quantities of initial loading amounts of RNA are difficult to accurately quantify.

**Figure 1:**
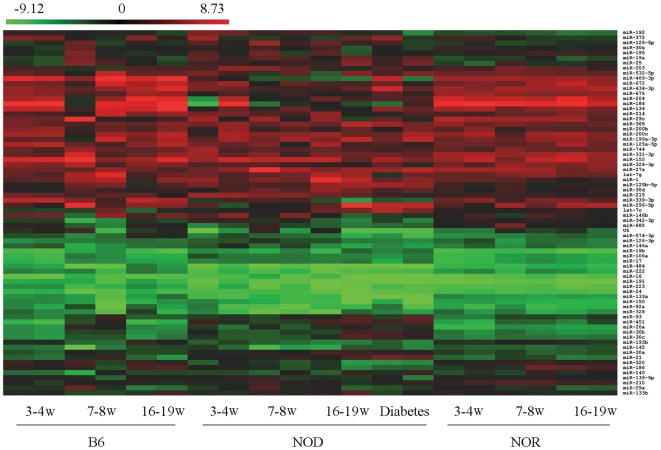
Global expression of serum miRNAs in different mouse strains. Serum RNA was isolated from different aged (w = weeks) mouse strains (n = 20), with each time point duplicated. The globally normalized ΔC_T_ values are represented in the heat map with miRNA species ordered by hierarchical clustering. miRNA expression was analyzed by TaqMan® miRNA arrays and normalized by StatMiner® global normalization.

Of the 277 individually expressed miRNAs, 72 displayed consistent expression across all 20 samples (Table S1). Using these 72 miRNAs as the global normalization reference set in Statminer®, a heat map of all expression values (−ΔC_T_) was generated (**Figure 1**). In order to be considered as potential endogenous references, only those miRNAs that had an ANOVA *p*>0.3 for the comparison of the B6, NOD, and NOR (n = 20) mouse strains were selected. This 3-group comparison provided 21 miRNAs with ANOVA *p*>0.3. The same criterion was applied to the pair-wise comparison of the 21 genes which produced 10 miRNAs with least-significant difference *p*>0.3. These 10 miRNAs passed our initial criteria of *p*>0.3 and were considered further (**Table 1**).

**Table 1:** Mouse endogenous control selection process.

Candidate miRNA	ANOVA			Pair-wise
	*p*>0.3	LSD *p*>0.3	SD<1	|ΔΔCt|<0.5
**miR-195**	0.9695646	pass	0.9910273	pass
**miR-30e**	0.9573852	pass	0.7163731	pass
**miR-146a**	0.9403384	pass	0.9486185	pass
**miR-16**	0.8665249	pass	0.8232968	pass
**miR-744**	0.5773991	pass	0.7078349	pass
**miR-29a**	0.8524583	pass	1.0477783	
**miR-200c**	0.7367455	pass	1.1338788	
**miR-let 7g**	0.7352368	pass	1.2810793	
**miR-214**	0.6059579	pass	1.2313915	
**miR-2965p**	0.9875817	pass	2.182499	
**miR-133b**	0.5180362	fail		
**miR-199a3p**	0.5143139	fail		
**miR-5325p**	0.4387915	fail		
**miR-146b**	0.4132751	fail		
**miR-145**	0.4100232	fail		
**miR-1395p**	0.3748676	fail		
**miR-200b**	0.373373	fail		
**miR-375**	0.3580763	fail		
**miR-150**	0.3283348	fail		
**miR-328**	0.3274329	fail		
**miR-92a**	0.3249034	fail		

From 335 potential target miRNAs, 277 total miRNAs were expressed over all samples, and 72 miRNAs were expressed in all 20 samples. These 72 miRNAs were candidate endogenous controls, following a series of statistical analyses only five genes (miR-146a, miR-16, miR-195, miR-30e, and miR-744) passed the criteria of ANOVA *p*>0.3, SD<1, and pair-wise |ΔΔ*C*_T_|<0.5.

**Figure 2:**
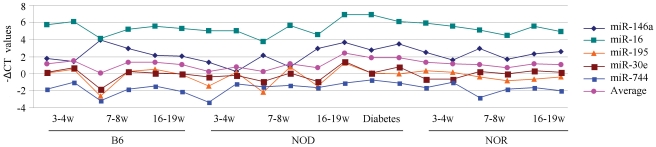
Five serum miRNAs as endogenous references stably expressed across different mouse strains. −ΔC_T_ values of miR-146a, miR-16, miR-195, miR-30e, and miR-744 show stability across all samples (w = weeks). The −ΔC_T_ average of all five miRNAs is also displayed. Each gene is expressed across all samples, has an ANOVA *p*>0.3, an SD<1 and all pair-wise |ΔΔC_T_|<0.5.

The second criterion necessary for endogenous reference consideration were miRNAs with a SD<1. This is similar to the criterion used to determine normalizers for miRNA expression in various tissues [22], [23]. As a result the SD values of the 10 candidate genes were calculated, and five miRNAs passed our criteria of a *p*>0.3, and an SD<1. Finally, the five miRNAs also met the criterion of all pair-wise |ΔΔC_T_|<0.5 (**Table 1**). As shown in **Figure 2**, these five serum miRNAs, miR-146a, miR-16, miR-195, miR-30e, and miR-744 were stably expressed in mouse regardless of strain, age, and disease condition. Furthermore, miR-146a and miR-16 are highly expressed in serum, while miR-30e, miR-195, and miR-744 have relatively lower expression.

### U6 is not stably expressed in mouse serum

**Figure 3:**
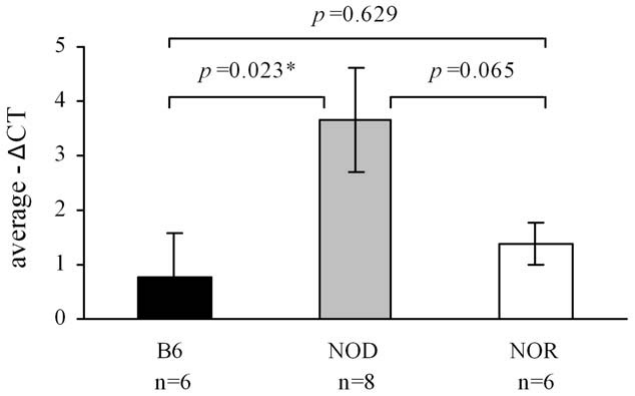
MammU6 is differentially expressed in the different mouse strains. Bar plots of the average MammU6 expression for each mouse strain are given with standard error bars. An ANOVA comparison detects a significant difference in the means, *p*-value of 0.049, an LSD *p*-value of 0.023 (B6 vs. NOD), and common SD of 2.4.

MammU6 (U6) RNA is commonly used as a normalizer for miRNA expression in tissues and cells [20], [24]. Recently, the use of this RNA has been extended to normalization of circulating miRNA biomarkers [25], [26], [27]. As shown in **Figure 3**, U6 is expressed in all 20 samples, however, failed to meet our criteria as an endogenous reference. Based on the global normalization, U6 displays an ANOVA *p*-value of 0.049 and an SD of 2.4, with at least a 4 fold difference in the expression between NOD vs. both B6 and NOR. Thus, U6 may not serve as a serum normalizer for miRNAs.

## Discussion

In the current study, we have defined five serum miRNAs as endogenous references. To our knowledge this is the first study of serum endogenous references for mouse miRNAs. Normalization techniques for circulating miRNAs have yet to be perfected. Some of the normalizing methods for serum or plasma miRNA reported are adopted from tissue studies, including the use of U6, which has stirred concern in recent studies. snoRNA and RNU6B (U6) have been described as instable in serum and therefore cannot be used for normalization [28]. U6 and 5S were reportedly degraded in some serum samples [15]. As reported in our current study, our data further confirmed that U6 is not a good candidate for an endogenous reference for serum miRNA expression. Additionally, miRNA miR-191 and miR-103 have been reported as internal controls for tissue miRNA expression [20]. Serum miR-191 is expressed in all samples, however there is a significant difference (*p* = 0.001) between all samples (data not shown here). Therefore, miR-191 fails to meet our endogenous control criteria and was not considered further as a serum miRNA internal reference. Similarly, serum miR-103 does not have the stability as reported in tissue samples, being only expressed in 5 of our 20 samples (data not shown here), and does not meet our endogenous control requirements either. Thus, tissue miRNA internal controls may not be suitable for serum internal references.

The total miRNAs present in serum and plasma are very low, and efficient reproducible recovery of this RNA is a challenging task. Furthermore, the yield of RNA from small volume serum or plasma samples has, in our hand and others, been below the limit of accurate quantitation by OD measurement. Considerable sample-to-sample (strain to strain in mouse) variability in both protein and lipid content of plasma and serum samples, which could potentially affect efficiency of RNA extraction and introduce inhibitors of PCR reactions, will further add to the variation of RNA extraction and efficiency of PCR reactions. In order to adjust for those variations, *C. elegans* synthetic miRNAs spiked in at the onset of the RNA isolation process has been adopted by many researchers to provide an internal reference for normalization of technical variations between samples [13]. However, variation of the amount of miRNAs present in a given individual serum or plasma sample, which could be genetically controlled or introduced by many factors, cannot be normalized by this strategy. Invariant serum housekeeping miRNAs are urgently needed for circulating miRNA studies [29]. Without an acceptable standard for normalization, circulating miRNA biomarkers are limited in claims of diagnostic discovery and evaluation because they cannot be evaluated individually.

It is expected, theoretically, that a true endogenous reference would be stably expressed, where expressions are not affected by the difference of strains, age, and conditions, including diseases. The five genes we identified here are an important discovery and may serve as good normalizers for circulating miRNAs for several reasons. First, 20 miRNA microarrays have been completed which are a large number of arrays from a variety of mouse strains and ages. Second, in addition to a low SD, a criterion successfully used to identify tissue endogenous references [22], [23], the additional criteria, ANOVA *p*>0.3 and pair-wise |ΔΔC_T_|<0.5, have also been employed in our strategy in order to strengthen our endogenous reference selection. Finally, the fact that consistent expression of five genes in circulating miRNA expression profiles exists from multiple mouse strains, at different ages, and with different disease models provides strong evidence that miR-146a, miR-16, miR-195, miR-30e, and miR-744 are useful as circulating miRNA endogenous references. Using a selection of these candidates, we have successfully validated some serum biomarkers for diabetes in NOD mice as well as metastatic lung melanoma in the mouse model by single qRT-PCR reactions following global normalization of array data (our unpublished data).

When choosing a qRT-PCR serum endogenous reference, we recommend careful consideration of endogenous miRNA controls based on RNA loading amounts and miRNA expression abundances. As shown in **Figure 2**, miR-146a and miR-16 are highly expressed in serum, while miR-30e, miR-195, and miR-744 have lower expression levels. For instance, if an experiment is working with lower abundance of miRNA, it may be worthwhile to select those controls with lower expression in order to properly validate the miRNA of interest. In addition, miR-146a has been implicated in immune cell regulation, cancer, β-cell failure, and potentially type 2 diabetes [29], [30], [31], [32]. Therefore, more precautions should be taken for the use of miR-146a as an endogenous reference in certain disease models. We preformed normalization of single qRT-PCR targets from the related mouse disease model using miR-146a, miR-16, and miR-195. Cross normalization of these references to each other revealed that miR-16 and miR-195 are extremely stable endogenous references in this particular mouse model (our unpublished data). We recommend that suitable endogenous controls should be selected in light of the study design and research conditions, and that the use of 2–3 endogenous references together may additionally reduce bias and variation [20], [30], [31]. Choosing more than one reference miRNA will reduce the possibility that any endogenous references may be regulated by specific conditions, in which these references should not serve as endogenous references. Small fold change differences of potential biomarkers may not be identified with our reference genes, however we are confident that based on the stability of the miRNAs presented here the biomarkers identified with these reference genes is better than currently accepted serum miRNA references.

Currently, there are no reliable human serum internal references reported. It is unclear whether the identified five miRNAs here are also stably expressed in human serum samples. Notably, these five miRNAs, miR-16, miR-744, miR-195, miR-146a, and miR-30e share a 100% identity between human and mouse [32], [33], [34], [35]. Interestingly, two recently published papers reporting on serum miRNA biomarkers for specific diseases have used miR-16 as an internal control to normalize qRT-PCR data [16], [36]. However, a large number of human samples are needed to further validate this endogenous reference.

In conclusion, using a TaqMan® real-time PCR miRNA array and global gene expression normalization strategy, we have analyzed 20 individual serum miRNA expression profiles from multiple animal strains across different ages as well as different conditions. We have found five miRNAs, miR-146a, miR-16, miR-195, miR-30e, and miR-744 to be stably expressed in all tested strains across different ages and conditions. Thus, these miRNAs may be used as mouse serum miRNA endogenous references necessary for single assay studies. The discovery of consistent expression of these specific serum miRNAs will promote the generation of more accurate miRNA expression profiles and more accurate interpretation of qRT-PCR data. Although we are convinced that these five miRNAs are the best circulating endogenous references reported so far, the question why these miRNAs are stable in mouse serum remains to be answered. Further investigation into the function of these specific serum miRNAs, the validation of their expression stability in different disease conditions, and their expression stability in human samples are needed.

## Supporting Information

Table S1 **Results of TaqMan® Low-Density Array miRNA qRT-PCR expression levels of B6, NOD, and NOR female mice.** C_T_ values of miRNA expressed in all samples. These miRNAs were considered as candidates for circulating endogenous control candidates. miRNA names and identification numbers are from the TaqMan® Rodent MicroRNA qRT-PCR array card A v2.0.
(DOC)

## References

[1] Bartel DP (2004). MicroRNAs: genomics, biogenesis, mechanism, and function.. Cell.

[2] Chen CZ, Lodish HF (2005). MicroRNAs as regulators of mammalian hematopoiesis.. Semin Immunol.

[3] Zhou L, Seo KH, Wong HK, Mi QS (2009). MicroRNAs and immune regulatory T cells.. Int Immunopharmacol.

[4] Zhou L, Seo KH, He HZ, Pacholczyk R, Meng DM (2009). Tie2cre-induced inactivation of the miRNA-processing enzyme Dicer disrupts invariant NKT cell development.. Proc Natl Acad Sci U S A.

[5] Cobb BS, Hertweck A, Smith J, O'Connor E, Graf D (2006). A role for Dicer in immune regulation.. J Exp Med.

[6] Calin GA, Croce CM (2006). MicroRNA signatures in human cancers.. Nat Rev Cancer.

[7] Esquela-Kerscher A, Slack FJ (2006). Oncomirs - microRNAs with a role in cancer.. Nat Rev Cancer.

[8] Mandel P, Metais P (1948). Les acides necleiques du plasma sanguin chez l'Homme.. C R Acad Sci Paris.

[9] Stroun M, Anker P, Maurice P, Lyautey J, Lederrey C (1989). Neoplastic characteristics of the DNA found in the plasma of cancer patients.. Oncology.

[10] Leon SA, Shapiro B, Sklaroff DM, Yaros MJ (1977). Free DNA in the serum of cancer patients and the effect of therapy.. Cancer Res.

[11] Tong YK, Lo YM (2006). Diagnostic developments involving cell-free (circulating) nucleic acids.. Clin Chim Acta.

[12] Gilad S, Meiri E, Yogev Y, Benjamin S, Lebanony D (2008). Serum microRNAs are promising novel biomarkers.. PLoS One.

[13] Mitchell PS, Parkin RK, Kroh EM, Fritz BR, Wyman SK (2008). Circulating microRNAs as stable blood-based markers for cancer detection.. Proc Natl Acad Sci U S A.

[14] Wang K, Zhang S, Marzolf B, Troisch P, Brightman A (2009). Circulating microRNAs, potential biomarkers for drug-induced liver injury.. Proc Natl Acad Sci U S A.

[15] Chen X, Ba Y, Ma L, Cai X, Yin Y (2008). Characterization of microRNAs in serum: a novel class of biomarkers for diagnosis of cancer and other diseases.. Cell Res.

[16] Lawrie CH, Gal S, Dunlop HM, Pushkaran B, Liggins AP (2008). Detection of elevated levels of tumour-associated microRNAs in serum of patients with diffuse large B-cell lymphoma.. Br J Haematol.

[17] Ji X, Takahashi R, Hiura Y, Hirokawa G, Fukushima Y (2009). Plasma miR-208 as a biomarker of myocardial injury.. Clin Chem.

[18] Laterza OF, Lim L, Garrett-Engele PW, Vlasakova K, Muniappa N (2009). Plasma MicroRNAs as sensitive and specific biomarkers of tissue injury.. Clin Chem.

[19] D'Alessandra Y, Devanna P, Limana F, Straino S, Di Carlo A (2010). Circulating microRNAs are new and sensitive biomarkers of myocardial infarction.. Eur Heart J.

[20] Peltier HJ, Latham GJ (2008). Normalization of microRNA expression levels in quantitative RT-PCR assays: identification of suitable reference RNA targets in normal and cancerous human solid tissues.. RNA.

[21] Cui W, Ma J, Wang Y, Biswal S (2011). Plasma miRNA as biomarkers for assessment of total-body radiation exposure dosimetry.. PLoS One.

[22] de Kok JB, Roelofs RW, Giesendorf BA, Pennings JL, Waas ET (2005). Normalization of gene expression measurements in tumor tissues: comparison of 13 endogenous control genes.. Lab Invest.

[23] Lossos IS, Czerwinski DK, Wechser MA, Levy R (2003). Optimization of quantitative real-time RT-PCR parameters for the study of lymphoid malignancies.. Leukemia.

[24] Shell S, Park SM, Radjabi AR, Schickel R, Kistner EO (2007). Let-7 expression defines two differentiation stages of cancer.. Proc Natl Acad Sci U S A.

[25] Ng EK, Chong WW, Jin H, Lam EK, Shin VY (2009). Differential expression of microRNAs in plasma of patients with colorectal cancer: a potential marker for colorectal cancer screening.. Gut.

[26] Vasilescu C, Rossi S, Shimizu M, Tudor S, Veronese A (2009). MicroRNA fingerprints identify miR-150 as a plasma prognostic marker in patients with sepsis.. PLoS One.

[27] Tsujiura M, Ichikawa D, Komatsu S, Shiozaki A, Takeshita H (2010). Circulating microRNAs in plasma of patients with gastric cancers.. Br J Cancer.

[28] Wittmann J, Jack HM (2010). Serum microRNAs as powerful cancer biomarkers.. Biochim Biophys Acta.

[29] Kroh EM, Parkin RK, Mitchell PS, Tewari M (2010). Analysis of circulating microRNA biomarkers in plasma and serum using quantitative reverse transcription-PCR (qRT-PCR).. Methods.

[30] Andersen CL, Jensen JL, Orntoft TF (2004). Normalization of real-time quantitative reverse transcription-PCR data: a model-based variance estimation approach to identify genes suited for normalization, applied to bladder and colon cancer data sets.. Cancer Res.

[31] Vandesompele J, De Preter K, Pattyn F, Poppe B, Van Roy N (2002). Accurate normalization of real-time quantitative RT-PCR data by geometric averaging of multiple internal control genes.. Genome Biol.

[32] Kozomara A, Griffiths-Jones S (2011). miRBase: integrating microRNA annotation and deep-sequencing data.. Nucleic Acids Res.

[33] Griffiths-Jones S, Saini HK, van Dongen S, Enright AJ (2008). miRBase: tools for microRNA genomics.. Nucleic Acids Res.

[34] Griffiths-Jones S, Grocock RJ, van Dongen S, Bateman A, Enright AJ (2006). miRBase: microRNA sequences, targets and gene nomenclature.. Nucleic Acids Res.

[35] Griffiths-Jones S (2004). The microRNA Registry.. Nucleic Acids Res.

[36] Huang Z, Huang D, Ni S, Peng Z, Sheng W (2010). Plasma microRNAs are promising novel biomarkers for early detection of colorectal cancer.. Int J Cancer.

