# Supplementary material for: Identification of Mouse Serum miRNA Endogenous References by Global Gene Expression Profiles
Source: PLoS One. 2012 Feb 10;7(2):e31278. doi: 10.1371/journal.pone.0031278 (PMC3277497; doi:10.1371/journal.pone.0031278)
Supplement: Table S1 — Results of TaqMan® Low-Density Array miRNA qRT-PCR expression levels of B6, NOD, and NOR female mice. CT values of miRNA expressed in all samples. These miRNAs were considered as candidates for circulating endogenous control candidates. miRNA names and identification numbers are from the TaqMan® Rodent MicroRNA qRT-PCR array card A v2.0. (DOC) [file pone.0031278.s001.doc]

**Table S1. Results of** **TaqMan® Low-Density Array miRNA qRT-PCR expression levels of B6, NOD, and NOR female mice**

| **Sample** | **Detector** | ***C*_T_** |
| --- | --- | --- |
| B6 3-4 wks 1 A | MammU6-4395470 | 19.10053 |
| B6 3-4 wks 1 A | MammU6-4395470 | 19.23702 |
| B6 3-4 wks 1 A | MammU6-4395470 | 19.30872 |
| B6 3-4 wks 1 A | MammU6-4395470 | 19.37106 |
| B6 3-4 wks 1 A | mmu-let-7c-4373167 | 18.96537 |
| B6 3-4 wks 1 A | mmu-let-7g-4395393 | 21.48999 |
| B6 3-4 wks 1 A | mmu-miR-106a-4395589 | 15.46047 |
| B6 3-4 wks 1 A | mmu-miR-125a-5p-4395309 | 23.53719 |
| B6 3-4 wks 1 A | mmu-miR-125b-5p-4373148 | 19.96419 |
| B6 3-4 wks 1 A | mmu-miR-126-3p-4395339 | 16.67837 |
| B6 3-4 wks 1 A | mmu-miR-126-5p-4373269 | 18.83587 |
| B6 3-4 wks 1 A | mmu-miR-133a-4395357 | 16.54263 |
| B6 3-4 wks 1 A | mmu-miR-133b-4395358 | 19.43784 |
| B6 3-4 wks 1 A | mmu-miR-134-4373299 | 24.00104 |
| B6 3-4 wks 1 A | mmu-miR-139-5p-4395400 | 19.8159 |
| B6 3-4 wks 1 A | mmu-miR-140-4373374 | 17.94125 |
| B6 3-4 wks 1 A | mmu-miR-1-4395333 | 21.00408 |
| B6 3-4 wks 1 A | mmu-miR-145-4395389 | 17.64989 |
| B6 3-4 wks 1 A | mmu-miR-146a-4373132 | 17.9618 |
| B6 3-4 wks 1 A | mmu-miR-146b-4373178 | 21.54716 |
| B6 3-4 wks 1 A | mmu-miR-150-4373127 | 17.05995 |
| B6 3-4 wks 1 A | mmu-miR-155-4395701 | 24.64557 |
| B6 3-4 wks 1 A | mmu-miR-16-4373121 | 13.95712 |
| B6 3-4 wks 1 A | mmu-miR-17-4395419 | 15.74734 |
| B6 3-4 wks 1 A | mmu-miR-184-4373113 | 26.96385 |
| B6 3-4 wks 1 A | mmu-miR-186-4395396 | 20.1521 |
| B6 3-4 wks 1 A | mmu-miR-191-4395410 | 15.8385 |
| B6 3-4 wks 1 A | mmu-miR-192-4373108 | 20.22944 |
| B6 3-4 wks 1 A | mmu-miR-193b-4395597 | 19.45211 |
| B6 3-4 wks 1 A | mmu-miR-195-4373105 | 19.69979 |
| B6 3-4 wks 1 A | mmu-miR-199a-3p-4395415 | 21.30368 |
| B6 3-4 wks 1 A | mmu-miR-19a-4373099 | 18.98008 |
| B6 3-4 wks 1 A | mmu-miR-19b-4373098 | 15.02127 |
| B6 3-4 wks 1 A | mmu-miR-200b-4395362 | 22.43065 |
| B6 3-4 wks 1 A | mmu-miR-200c-4395411 | 23.37013 |
| B6 3-4 wks 1 A | mmu-miR-203-4373095 | 20.72256 |
| B6 3-4 wks 1 A | mmu-miR-204-4373094 | 23.39448 |
| B6 3-4 wks 1 A | mmu-miR-20a-4373286 | 16.94237 |
| B6 3-4 wks 1 A | mmu-miR-210-4373089 | 19.75853 |
| B6 3-4 wks 1 A | mmu-miR-21-4373090 | 18.01956 |
| B6 3-4 wks 1 A | mmu-miR-214-4395417 | 20.98926 |
| B6 3-4 wks 1 A | mmu-miR-215-4373316 | 21.85247 |
| B6 3-4 wks 1 A | mmu-miR-222-4395387 | 17.24063 |
| B6 3-4 wks 1 A | mmu-miR-223-4395406 | 16.77151 |
| B6 3-4 wks 1 A | mmu-miR-24-4373072 | 15.53819 |
| B6 3-4 wks 1 A | mmu-miR-25-4373071 | 19.19149 |
| B6 3-4 wks 1 A | mmu-miR-27a-4373287 | 21.84278 |
| B6 3-4 wks 1 A | mmu-miR-296-5p-4373066 | 20.91049 |
| B6 3-4 wks 1 A | mmu-miR-29a-4395223 | 19.0961 |
| B6 3-4 wks 1 A | mmu-miR-29c-4395171 | 21.98913 |
| B6 3-4 wks 1 A | mmu-miR-30a-4373061 | 18.52377 |
| B6 3-4 wks 1 A | mmu-miR-30b-4373290 | 16.96602 |
| B6 3-4 wks 1 A | mmu-miR-30c-4373060 | 16.92822 |
| B6 3-4 wks 1 A | mmu-miR-30d-4373059 | 19.95046 |
| B6 3-4 wks 1 A | mmu-miR-30e-4395334 | 19.56504 |
| B6 3-4 wks 1 A | mmu-miR-320-4395388 | 20.49139 |
| B6 3-4 wks 1 A | mmu-miR-324-3p-4395639 | 22.65394 |
| **Sample** | **Detector** | ***C*_T_** |
| B6 3-4 wks 1 A | mmu-miR-328-4373049 | 17.87785 |
| B6 3-4 wks 1 A | mmu-miR-331-3p-4373046 | 21.49976 |
| B6 3-4 wks 1 A | mmu-miR-339-3p-4395663 | 23.77786 |
| B6 3-4 wks 1 A | mmu-miR-342-3p-4395371 | 18.82648 |
| B6 3-4 wks 1 A | mmu-miR-365-4373194 | 21.99343 |
| B6 3-4 wks 1 A | mmu-miR-375-4373027 | 22.36434 |
| B6 3-4 wks 1 A | mmu-miR-409-3p-4395443 | 25.27114 |
| B6 3-4 wks 1 A | mmu-miR-434-3p-4395734 | 22.95705 |
| B6 3-4 wks 1 A | mmu-miR-451-4373360 | 15.57967 |
| B6 3-4 wks 1 A | mmu-miR-484-4381032 | 16.3602 |
| B6 3-4 wks 1 A | mmu-miR-532-5p-4380928 | 21.37228 |
| B6 3-4 wks 1 A | mmu-miR-574-3p-4395460 | 18.53567 |
| B6 3-4 wks 1 A | mmu-miR-672-4395438 | 21.96506 |
| B6 3-4 wks 1 A | mmu-miR-676-4386776 | 22.84346 |
| B6 3-4 wks 1 A | mmu-miR-685-4386748 | 20.02285 |
| B6 3-4 wks 1 A | mmu-miR-744-4395435 | 21.52641 |
| B6 3-4 wks 1 A | mmu-miR-92a-4373013 | 17.01923 |
| B6 3-4 wks 1 A | mmu-miR-93-4373302 | 17.63422 |
| B6 3-4 wks 2 A | MammU6-4395470 | 19.82493 |
| B6 3-4 wks 2 A | MammU6-4395470 | 19.87732 |
| B6 3-4 wks 2 A | MammU6-4395470 | 20.03725 |
| B6 3-4 wks 2 A | MammU6-4395470 | 20.13094 |
| B6 3-4 wks 2 A | mmu-let-7c-4373167 | 18.8175 |
| B6 3-4 wks 2 A | mmu-let-7g-4395393 | 20.94472 |
| B6 3-4 wks 2 A | mmu-miR-106a-4395589 | 15.12119 |
| B6 3-4 wks 2 A | mmu-miR-125a-5p-4395309 | 23.92711 |
| B6 3-4 wks 2 A | mmu-miR-125b-5p-4373148 | 19.89899 |
| B6 3-4 wks 2 A | mmu-miR-126-3p-4395339 | 17.16487 |
| B6 3-4 wks 2 A | mmu-miR-126-5p-4373269 | 19.41483 |
| B6 3-4 wks 2 A | mmu-miR-133a-4395357 | 16.98452 |
| B6 3-4 wks 2 A | mmu-miR-133b-4395358 | 19.92044 |
| B6 3-4 wks 2 A | mmu-miR-134-4373299 | 24.09738 |
| B6 3-4 wks 2 A | mmu-miR-139-5p-4395400 | 19.94848 |
| B6 3-4 wks 2 A | mmu-miR-140-4373374 | 17.6986 |
| B6 3-4 wks 2 A | mmu-miR-1-4395333 | 21.46958 |
| B6 3-4 wks 2 A | mmu-miR-145-4395389 | 17.46553 |
| B6 3-4 wks 2 A | mmu-miR-146a-4373132 | 18.32058 |
| B6 3-4 wks 2 A | mmu-miR-146b-4373178 | 21.96524 |
| B6 3-4 wks 2 A | mmu-miR-150-4373127 | 16.97136 |
| B6 3-4 wks 2 A | mmu-miR-155-4395701 | 24.96114 |
| B6 3-4 wks 2 A | mmu-miR-16-4373121 | 13.60256 |
| B6 3-4 wks 2 A | mmu-miR-17-4395419 | 15.26529 |
| B6 3-4 wks 2 A | mmu-miR-184-4373113 | 27.1913 |
| B6 3-4 wks 2 A | mmu-miR-186-4395396 | 19.97476 |
| B6 3-4 wks 2 A | mmu-miR-191-4395410 | 15.9675 |
| B6 3-4 wks 2 A | mmu-miR-192-4373108 | 20.19096 |
| B6 3-4 wks 2 A | mmu-miR-193b-4395597 | 19.48888 |
| B6 3-4 wks 2 A | mmu-miR-195-4373105 | 19.22349 |
| B6 3-4 wks 2 A | mmu-miR-199a-3p-4395415 | 21.37599 |
| B6 3-4 wks 2 A | mmu-miR-19a-4373099 | 18.43499 |
| B6 3-4 wks 2 A | mmu-miR-19b-4373098 | 14.76822 |
| B6 3-4 wks 2 A | mmu-miR-200b-4395362 | 22.9312 |
| B6 3-4 wks 2 A | mmu-miR-200c-4395411 | 23.27612 |
| B6 3-4 wks 2 A | mmu-miR-203-4373095 | 20.70803 |
| B6 3-4 wks 2 A | mmu-miR-204-4373094 | 23.82391 |
| B6 3-4 wks 2 A | mmu-miR-20a-4373286 | 16.40537 |
| B6 3-4 wks 2 A | mmu-miR-210-4373089 | 18.96896 |
| B6 3-4 wks 2 A | mmu-miR-21-4373090 | 17.60817 |
| B6 3-4 wks 2 A | mmu-miR-214-4395417 | 20.6508 |
| B6 3-4 wks 2 A | mmu-miR-215-4373316 | 21.28828 |
| B6 3-4 wks 2 A | mmu-miR-222-4395387 | 16.96436 |
| **Sample** | **Detector** | ***C*_T_** |
| B6 3-4 wks 2 A | mmu-miR-223-4395406 | 16.79855 |
| B6 3-4 wks 2 A | mmu-miR-24-4373072 | 15.7491 |
| B6 3-4 wks 2 A | mmu-miR-25-4373071 | 18.4727 |
| B6 3-4 wks 2 A | mmu-miR-27a-4373287 | 21.79297 |
| B6 3-4 wks 2 A | mmu-miR-296-5p-4373066 | 19.97621 |
| B6 3-4 wks 2 A | mmu-miR-29a-4395223 | 19.29928 |
| B6 3-4 wks 2 A | mmu-miR-29c-4395171 | 21.70655 |
| B6 3-4 wks 2 A | mmu-miR-30a-4373061 | 17.96005 |
| B6 3-4 wks 2 A | mmu-miR-30b-4373290 | 16.46668 |
| B6 3-4 wks 2 A | mmu-miR-30c-4373060 | 16.27122 |
| B6 3-4 wks 2 A | mmu-miR-30d-4373059 | 19.58257 |
| B6 3-4 wks 2 A | mmu-miR-30e-4395334 | 19.04738 |
| B6 3-4 wks 2 A | mmu-miR-320-4395388 | 19.95357 |
| B6 3-4 wks 2 A | mmu-miR-324-3p-4395639 | 22.06744 |
| B6 3-4 wks 2 A | mmu-miR-328-4373049 | 17.00038 |
| B6 3-4 wks 2 A | mmu-miR-331-3p-4373046 | 21.11178 |
| B6 3-4 wks 2 A | mmu-miR-339-3p-4395663 | 23.96036 |
| B6 3-4 wks 2 A | mmu-miR-342-3p-4395371 | 18.96444 |
| B6 3-4 wks 2 A | mmu-miR-365-4373194 | 21.53272 |
| B6 3-4 wks 2 A | mmu-miR-375-4373027 | 22.18203 |
| B6 3-4 wks 2 A | mmu-miR-409-3p-4395443 | 24.85479 |
| B6 3-4 wks 2 A | mmu-miR-434-3p-4395734 | 22.34473 |
| B6 3-4 wks 2 A | mmu-miR-451-4373360 | 14.96393 |
| B6 3-4 wks 2 A | mmu-miR-484-4381032 | 15.97696 |
| B6 3-4 wks 2 A | mmu-miR-532-5p-4380928 | 21.05037 |
| B6 3-4 wks 2 A | mmu-miR-574-3p-4395460 | 18.46899 |
| B6 3-4 wks 2 A | mmu-miR-672-4395438 | 21.80534 |
| B6 3-4 wks 2 A | mmu-miR-676-4386776 | 22.61075 |
| B6 3-4 wks 2 A | mmu-miR-685-4386748 | 19.97051 |
| B6 3-4 wks 2 A | mmu-miR-744-4395435 | 20.80067 |
| B6 3-4 wks 2 A | mmu-miR-92a-4373013 | 15.95016 |
| B6 3-4 wks 2 A | mmu-miR-93-4373302 | 16.94484 |
| B6 7-8 wks 1 A | MammU6-4395470 | 30.70337 |
| B6 7-8 wks 1 A | MammU6-4395470 | 30.94963 |
| B6 7-8 wks 1 A | MammU6-4395470 | 30.96356 |
| B6 7-8 wks 1 A | MammU6-4395470 | 31.48008 |
| B6 7-8 wks 1 A | mmu-let-7c-4373167 | 29.06169 |
| B6 7-8 wks 1 A | mmu-let-7g-4395393 | 33.83202 |
| B6 7-8 wks 1 A | mmu-miR-106a-4395589 | 27.66787 |
| B6 7-8 wks 1 A | mmu-miR-125a-5p-4395309 | 31.79842 |
| B6 7-8 wks 1 A | mmu-miR-125b-5p-4373148 | 31.94272 |
| B6 7-8 wks 1 A | mmu-miR-126-3p-4395339 | 27.52829 |
| B6 7-8 wks 1 A | mmu-miR-126-5p-4373269 | 31.97832 |
| B6 7-8 wks 1 A | mmu-miR-133a-4395357 | 26.53858 |
| B6 7-8 wks 1 A | mmu-miR-133b-4395358 | 28.96797 |
| B6 7-8 wks 1 A | mmu-miR-134-4373299 | 30.69537 |
| B6 7-8 wks 1 A | mmu-miR-139-5p-4395400 | 29.73072 |
| B6 7-8 wks 1 A | mmu-miR-140-4373374 | 26.04611 |
| B6 7-8 wks 1 A | mmu-miR-1-4395333 | 33.34637 |
| B6 7-8 wks 1 A | mmu-miR-145-4395389 | 23.36615 |
| B6 7-8 wks 1 A | mmu-miR-146a-4373132 | 26.04628 |
| B6 7-8 wks 1 A | mmu-miR-146b-4373178 | 27.9874 |
| B6 7-8 wks 1 A | mmu-miR-150-4373127 | 26.51942 |
| B6 7-8 wks 1 A | mmu-miR-155-4395701 | 36.86066 |
| B6 7-8 wks 1 A | mmu-miR-16-4373121 | 25.87213 |
| B6 7-8 wks 1 A | mmu-miR-17-4395419 | 27.18514 |
| B6 7-8 wks 1 A | mmu-miR-184-4373113 | 29.56124 |
| B6 7-8 wks 1 A | mmu-miR-186-4395396 | 30.57876 |
| B6 7-8 wks 1 A | mmu-miR-191-4395410 | 26.24304 |
| B6 7-8 wks 1 A | mmu-miR-192-4373108 | 30.03382 |
| B6 7-8 wks 1 A | mmu-miR-193b-4395597 | 28.07021 |
| **Sample** | **Detector** | ***C*_T_** |
| B6 7-8 wks 1 A | mmu-miR-195-4373105 | 32.58306 |
| B6 7-8 wks 1 A | mmu-miR-199a-3p-4395415 | 29.61033 |
| B6 7-8 wks 1 A | mmu-miR-19a-4373099 | 32.26913 |
| B6 7-8 wks 1 A | mmu-miR-19b-4373098 | 26.26427 |
| B6 7-8 wks 1 A | mmu-miR-200b-4395362 | 30.58134 |
| B6 7-8 wks 1 A | mmu-miR-200c-4395411 | 30.20682 |
| B6 7-8 wks 1 A | mmu-miR-203-4373095 | 29.54106 |
| B6 7-8 wks 1 A | mmu-miR-204-4373094 | 29.50426 |
| B6 7-8 wks 1 A | mmu-miR-20a-4373286 | 30.5083 |
| B6 7-8 wks 1 A | mmu-miR-210-4373089 | 30.04999 |
| B6 7-8 wks 1 A | mmu-miR-21-4373090 | 29.62111 |
| B6 7-8 wks 1 A | mmu-miR-214-4395417 | 29.84311 |
| B6 7-8 wks 1 A | mmu-miR-215-4373316 | 30.90929 |
| B6 7-8 wks 1 A | mmu-miR-222-4395387 | 27.52731 |
| B6 7-8 wks 1 A | mmu-miR-223-4395406 | 25.3572 |
| B6 7-8 wks 1 A | mmu-miR-24-4373072 | 24.0511 |
| B6 7-8 wks 1 A | mmu-miR-25-4373071 | 32.58962 |
| B6 7-8 wks 1 A | mmu-miR-27a-4373287 | 31.73644 |
| B6 7-8 wks 1 A | mmu-miR-296-5p-4373066 | 35.89776 |
| B6 7-8 wks 1 A | mmu-miR-29a-4395223 | 31.80747 |
| B6 7-8 wks 1 A | mmu-miR-29c-4395171 | 37.01256 |
| B6 7-8 wks 1 A | mmu-miR-30a-4373061 | 30.03311 |
| B6 7-8 wks 1 A | mmu-miR-30b-4373290 | 29.00807 |
| B6 7-8 wks 1 A | mmu-miR-30c-4373060 | 28.62766 |
| B6 7-8 wks 1 A | mmu-miR-30d-4373059 | 31.40308 |
| B6 7-8 wks 1 A | mmu-miR-30e-4395334 | 31.8392 |
| B6 7-8 wks 1 A | mmu-miR-320-4395388 | 31.13665 |
| B6 7-8 wks 1 A | mmu-miR-324-3p-4395639 | 34.00396 |
| B6 7-8 wks 1 A | mmu-miR-328-4373049 | 27.09598 |
| B6 7-8 wks 1 A | mmu-miR-331-3p-4373046 | 34.98602 |
| B6 7-8 wks 1 A | mmu-miR-339-3p-4395663 | 32.67032 |
| B6 7-8 wks 1 A | mmu-miR-342-3p-4395371 | 25.7067 |
| B6 7-8 wks 1 A | mmu-miR-365-4373194 | 31.01819 |
| B6 7-8 wks 1 A | mmu-miR-375-4373027 | 29.94434 |
| B6 7-8 wks 1 A | mmu-miR-409-3p-4395443 | 32.70865 |
| B6 7-8 wks 1 A | mmu-miR-434-3p-4395734 | 31.50589 |
| B6 7-8 wks 1 A | mmu-miR-451-4373360 | 28.64414 |
| B6 7-8 wks 1 A | mmu-miR-484-4381032 | 23.70694 |
| B6 7-8 wks 1 A | mmu-miR-532-5p-4380928 | 29.99744 |
| B6 7-8 wks 1 A | mmu-miR-574-3p-4395460 | 27.05901 |
| B6 7-8 wks 1 A | mmu-miR-672-4395438 | 30.5268 |
| B6 7-8 wks 1 A | mmu-miR-676-4386776 | 30.97866 |
| B6 7-8 wks 1 A | mmu-miR-685-4386748 | 28.49256 |
| B6 7-8 wks 1 A | mmu-miR-744-4395435 | 33.17763 |
| B6 7-8 wks 1 A | mmu-miR-92a-4373013 | 28.83914 |
| B6 7-8 wks 1 A | mmu-miR-93-4373302 | 28.97822 |
| B6 7-8 wks 2 A | MammU6-4395470 | 22.34959 |
| B6 7-8 wks 2 A | MammU6-4395470 | 22.361 |
| B6 7-8 wks 2 A | MammU6-4395470 | 22.42739 |
| B6 7-8 wks 2 A | MammU6-4395470 | 22.49122 |
| B6 7-8 wks 2 A | mmu-let-7c-4373167 | 28.9681 |
| B6 7-8 wks 2 A | mmu-let-7g-4395393 | 32.46151 |
| B6 7-8 wks 2 A | mmu-miR-106a-4395589 | 24.01093 |
| B6 7-8 wks 2 A | mmu-miR-125a-5p-4395309 | 27.78601 |
| B6 7-8 wks 2 A | mmu-miR-125b-5p-4373148 | 29.38002 |
| B6 7-8 wks 2 A | mmu-miR-126-3p-4395339 | 24.76743 |
| B6 7-8 wks 2 A | mmu-miR-126-5p-4373269 | 26.08345 |
| B6 7-8 wks 2 A | mmu-miR-133a-4395357 | 21.50582 |
| B6 7-8 wks 2 A | mmu-miR-133b-4395358 | 25.94134 |
| B6 7-8 wks 2 A | mmu-miR-134-4373299 | 31.80769 |
| B6 7-8 wks 2 A | mmu-miR-139-5p-4395400 | 25.21256 |
| **Sample** | **Detector** | ***C*_T_** |
| B6 7-8 wks 2 A | mmu-miR-140-4373374 | 26.90453 |
| B6 7-8 wks 2 A | mmu-miR-1-4395333 | 30.78053 |
| B6 7-8 wks 2 A | mmu-miR-145-4395389 | 24.49299 |
| B6 7-8 wks 2 A | mmu-miR-146a-4373132 | 24.01031 |
| B6 7-8 wks 2 A | mmu-miR-146b-4373178 | 29.86175 |
| B6 7-8 wks 2 A | mmu-miR-150-4373127 | 20.96525 |
| B6 7-8 wks 2 A | mmu-miR-155-4395701 | 29.62549 |
| B6 7-8 wks 2 A | mmu-miR-16-4373121 | 21.71796 |
| B6 7-8 wks 2 A | mmu-miR-17-4395419 | 23.98189 |
| B6 7-8 wks 2 A | mmu-miR-184-4373113 | 32.41253 |
| B6 7-8 wks 2 A | mmu-miR-186-4395396 | 25.61849 |
| B6 7-8 wks 2 A | mmu-miR-191-4395410 | 21.69927 |
| B6 7-8 wks 2 A | mmu-miR-192-4373108 | 26.64857 |
| B6 7-8 wks 2 A | mmu-miR-193b-4395597 | 24.94796 |
| B6 7-8 wks 2 A | mmu-miR-195-4373105 | 26.64067 |
| B6 7-8 wks 2 A | mmu-miR-199a-3p-4395415 | 31.73337 |
| B6 7-8 wks 2 A | mmu-miR-19a-4373099 | 27.44058 |
| B6 7-8 wks 2 A | mmu-miR-19b-4373098 | 23.9702 |
| B6 7-8 wks 2 A | mmu-miR-200b-4395362 | 28.59008 |
| B6 7-8 wks 2 A | mmu-miR-200c-4395411 | 30.47233 |
| B6 7-8 wks 2 A | mmu-miR-203-4373095 | 26.11834 |
| B6 7-8 wks 2 A | mmu-miR-204-4373094 | 29.9743 |
| B6 7-8 wks 2 A | mmu-miR-20a-4373286 | 26.92746 |
| B6 7-8 wks 2 A | mmu-miR-210-4373089 | 26.96289 |
| B6 7-8 wks 2 A | mmu-miR-21-4373090 | 27.53254 |
| B6 7-8 wks 2 A | mmu-miR-214-4395417 | 31.68995 |
| B6 7-8 wks 2 A | mmu-miR-215-4373316 | 28.54407 |
| B6 7-8 wks 2 A | mmu-miR-222-4395387 | 22.47713 |
| B6 7-8 wks 2 A | mmu-miR-223-4395406 | 22.75652 |
| B6 7-8 wks 2 A | mmu-miR-24-4373072 | 21.40721 |
| B6 7-8 wks 2 A | mmu-miR-25-4373071 | 28.18949 |
| B6 7-8 wks 2 A | mmu-miR-27a-4373287 | 30.16391 |
| B6 7-8 wks 2 A | mmu-miR-296-5p-4373066 | 28.08747 |
| B6 7-8 wks 2 A | mmu-miR-29a-4395223 | 26.4263 |
| B6 7-8 wks 2 A | mmu-miR-29c-4395171 | 27.25164 |
| B6 7-8 wks 2 A | mmu-miR-30a-4373061 | 26.64403 |
| B6 7-8 wks 2 A | mmu-miR-30b-4373290 | 26.06795 |
| B6 7-8 wks 2 A | mmu-miR-30c-4373060 | 25.66724 |
| B6 7-8 wks 2 A | mmu-miR-30d-4373059 | 27.60831 |
| B6 7-8 wks 2 A | mmu-miR-30e-4395334 | 26.69897 |
| B6 7-8 wks 2 A | mmu-miR-320-4395388 | 26.03526 |
| B6 7-8 wks 2 A | mmu-miR-324-3p-4395639 | 27.99444 |
| B6 7-8 wks 2 A | mmu-miR-328-4373049 | 21.5634 |
| B6 7-8 wks 2 A | mmu-miR-331-3p-4373046 | 27.9934 |
| B6 7-8 wks 2 A | mmu-miR-339-3p-4395663 | 27.7493 |
| B6 7-8 wks 2 A | mmu-miR-342-3p-4395371 | 24.02428 |
| B6 7-8 wks 2 A | mmu-miR-365-4373194 | 29.02011 |
| B6 7-8 wks 2 A | mmu-miR-375-4373027 | 26.38128 |
| B6 7-8 wks 2 A | mmu-miR-409-3p-4395443 | 34.79573 |
| B6 7-8 wks 2 A | mmu-miR-434-3p-4395734 | 30.00233 |
| B6 7-8 wks 2 A | mmu-miR-451-4373360 | 27.1178 |
| B6 7-8 wks 2 A | mmu-miR-484-4381032 | 21.13063 |
| B6 7-8 wks 2 A | mmu-miR-532-5p-4380928 | 31.51023 |
| B6 7-8 wks 2 A | mmu-miR-574-3p-4395460 | 24.96422 |
| B6 7-8 wks 2 A | mmu-miR-672-4395438 | 31.92304 |
| B6 7-8 wks 2 A | mmu-miR-676-4386776 | 28.23568 |
| B6 7-8 wks 2 A | mmu-miR-685-4386748 | 24.18817 |
| B6 7-8 wks 2 A | mmu-miR-744-4395435 | 28.7757 |
| B6 7-8 wks 2 A | mmu-miR-92a-4373013 | 20.69446 |
| B6 7-8 wks 2 A | mmu-miR-93-4373302 | 27.56195 |
| B6 16-19 wks 1 A | MammU6-4395470 | 17.77743 |
| **Sample** | **Detector** | ***C*_T_** |
| B6 16-19 wks 1 A | MammU6-4395470 | 18.02787 |
| B6 16-19 wks 1 A | MammU6-4395470 | 18.25164 |
| B6 16-19 wks 1 A | MammU6-4395470 | 18.25992 |
| B6 16-19 wks 1 A | mmu-let-7c-4373167 | 17.17072 |
| B6 16-19 wks 1 A | mmu-let-7g-4395393 | 19.79218 |
| B6 16-19 wks 1 A | mmu-miR-106a-4395589 | 14.95908 |
| B6 16-19 wks 1 A | mmu-miR-125a-5p-4395309 | 21.62786 |
| B6 16-19 wks 1 A | mmu-miR-125b-5p-4373148 | 18.44714 |
| B6 16-19 wks 1 A | mmu-miR-126-3p-4395339 | 16.96775 |
| B6 16-19 wks 1 A | mmu-miR-126-5p-4373269 | 19.14036 |
| B6 16-19 wks 1 A | mmu-miR-133a-4395357 | 15.47133 |
| B6 16-19 wks 1 A | mmu-miR-133b-4395358 | 18.33654 |
| B6 16-19 wks 1 A | mmu-miR-134-4373299 | 23.72641 |
| B6 16-19 wks 1 A | mmu-miR-139-5p-4395400 | 18.80481 |
| B6 16-19 wks 1 A | mmu-miR-140-4373374 | 17.93309 |
| B6 16-19 wks 1 A | mmu-miR-1-4395333 | 19.5478 |
| B6 16-19 wks 1 A | mmu-miR-145-4395389 | 17.77764 |
| B6 16-19 wks 1 A | mmu-miR-146a-4373132 | 16.71534 |
| B6 16-19 wks 1 A | mmu-miR-146b-4373178 | 19.93454 |
| B6 16-19 wks 1 A | mmu-miR-150-4373127 | 14.81634 |
| B6 16-19 wks 1 A | mmu-miR-155-4395701 | 22.21905 |
| B6 16-19 wks 1 A | mmu-miR-16-4373121 | 13.32084 |
| B6 16-19 wks 1 A | mmu-miR-17-4395419 | 15.37275 |
| B6 16-19 wks 1 A | mmu-miR-184-4373113 | 23.97526 |
| B6 16-19 wks 1 A | mmu-miR-186-4395396 | 19.91184 |
| B6 16-19 wks 1 A | mmu-miR-191-4395410 | 15.32423 |
| B6 16-19 wks 1 A | mmu-miR-192-4373108 | 18.62719 |
| B6 16-19 wks 1 A | mmu-miR-193b-4395597 | 19.57399 |
| B6 16-19 wks 1 A | mmu-miR-195-4373105 | 18.38846 |
| B6 16-19 wks 1 A | mmu-miR-199a-3p-4395415 | 20.98775 |
| B6 16-19 wks 1 A | mmu-miR-19a-4373099 | 18.70376 |
| B6 16-19 wks 1 A | mmu-miR-19b-4373098 | 14.37112 |
| B6 16-19 wks 1 A | mmu-miR-200b-4395362 | 20.04735 |
| B6 16-19 wks 1 A | mmu-miR-200c-4395411 | 21.70406 |
| B6 16-19 wks 1 A | mmu-miR-203-4373095 | 20.13618 |
| B6 16-19 wks 1 A | mmu-miR-204-4373094 | 24.83534 |
| B6 16-19 wks 1 A | mmu-miR-20a-4373286 | 16.2361 |
| B6 16-19 wks 1 A | mmu-miR-210-4373089 | 19.25295 |
| B6 16-19 wks 1 A | mmu-miR-21-4373090 | 17.0003 |
| B6 16-19 wks 1 A | mmu-miR-214-4395417 | 21.34661 |
| B6 16-19 wks 1 A | mmu-miR-215-4373316 | 20.43377 |
| B6 16-19 wks 1 A | mmu-miR-222-4395387 | 16.67315 |
| B6 16-19 wks 1 A | mmu-miR-223-4395406 | 15.9759 |
| B6 16-19 wks 1 A | mmu-miR-24-4373072 | 14.99135 |
| B6 16-19 wks 1 A | mmu-miR-25-4373071 | 18.95703 |
| B6 16-19 wks 1 A | mmu-miR-27a-4373287 | 20.95066 |
| B6 16-19 wks 1 A | mmu-miR-296-5p-4373066 | 21.62623 |
| B6 16-19 wks 1 A | mmu-miR-29a-4395223 | 16.38506 |
| B6 16-19 wks 1 A | mmu-miR-29c-4395171 | 18.81283 |
| B6 16-19 wks 1 A | mmu-miR-30a-4373061 | 17.81717 |
| B6 16-19 wks 1 A | mmu-miR-30b-4373290 | 15.8641 |
| B6 16-19 wks 1 A | mmu-miR-30c-4373060 | 15.60689 |
| B6 16-19 wks 1 A | mmu-miR-30d-4373059 | 19.17833 |
| B6 16-19 wks 1 A | mmu-miR-30e-4395334 | 18.81821 |
| B6 16-19 wks 1 A | mmu-miR-320-4395388 | 19.51222 |
| B6 16-19 wks 1 A | mmu-miR-324-3p-4395639 | 21.55126 |
| B6 16-19 wks 1 A | mmu-miR-328-4373049 | 18.13472 |
| B6 16-19 wks 1 A | mmu-miR-331-3p-4373046 | 20.69876 |
| B6 16-19 wks 1 A | mmu-miR-339-3p-4395663 | 23.26555 |
| B6 16-19 wks 1 A | mmu-miR-342-3p-4395371 | 18.45725 |
| B6 16-19 wks 1 A | mmu-miR-365-4373194 | 20.5183 |
| **Sample** | **Detector** | ***C*_T_** |
| B6 16-19 wks 1 A | mmu-miR-375-4373027 | 21.5772 |
| B6 16-19 wks 1 A | mmu-miR-409-3p-4395443 | 23.92726 |
| B6 16-19 wks 1 A | mmu-miR-434-3p-4395734 | 22.95703 |
| B6 16-19 wks 1 A | mmu-miR-451-4373360 | 14.34813 |
| B6 16-19 wks 1 A | mmu-miR-484-4381032 | 16.59213 |
| B6 16-19 wks 1 A | mmu-miR-532-5p-4380928 | 20.41713 |
| B6 16-19 wks 1 A | mmu-miR-574-3p-4395460 | 19.13523 |
| B6 16-19 wks 1 A | mmu-miR-672-4395438 | 22.63099 |
| B6 16-19 wks 1 A | mmu-miR-676-4386776 | 22.12603 |
| B6 16-19 wks 1 A | mmu-miR-685-4386748 | 19.30608 |
| B6 16-19 wks 1 A | mmu-miR-744-4395435 | 20.38158 |
| B6 16-19 wks 1 A | mmu-miR-92a-4373013 | 17.1606 |
| B6 16-19 wks 1 A | mmu-miR-93-4373302 | 17.25591 |
| B6 16-19 wks 2 A | MammU6-4395470 | 20.55189 |
| B6 16-19 wks 2 A | MammU6-4395470 | 20.60809 |
| B6 16-19 wks 2 A | MammU6-4395470 | 20.76899 |
| B6 16-19 wks 2 A | MammU6-4395470 | 20.78984 |
| B6 16-19 wks 2 A | mmu-let-7c-4373167 | 20.5669 |
| B6 16-19 wks 2 A | mmu-let-7g-4395393 | 22.99935 |
| B6 16-19 wks 2 A | mmu-miR-106a-4395589 | 16.95908 |
| B6 16-19 wks 2 A | mmu-miR-125a-5p-4395309 | 24.98436 |
| B6 16-19 wks 2 A | mmu-miR-125b-5p-4373148 | 22.17357 |
| B6 16-19 wks 2 A | mmu-miR-126-3p-4395339 | 18.73083 |
| B6 16-19 wks 2 A | mmu-miR-126-5p-4373269 | 20.40792 |
| B6 16-19 wks 2 A | mmu-miR-133a-4395357 | 18.87159 |
| B6 16-19 wks 2 A | mmu-miR-133b-4395358 | 21.87946 |
| B6 16-19 wks 2 A | mmu-miR-134-4373299 | 27.97942 |
| B6 16-19 wks 2 A | mmu-miR-139-5p-4395400 | 20.80769 |
| B6 16-19 wks 2 A | mmu-miR-140-4373374 | 20.00364 |
| B6 16-19 wks 2 A | mmu-miR-1-4395333 | 23.7836 |
| B6 16-19 wks 2 A | mmu-miR-145-4395389 | 19.95162 |
| B6 16-19 wks 2 A | mmu-miR-146a-4373132 | 18.84348 |
| B6 16-19 wks 2 A | mmu-miR-146b-4373178 | 23.21412 |
| B6 16-19 wks 2 A | mmu-miR-150-4373127 | 17.81768 |
| B6 16-19 wks 2 A | mmu-miR-155-4395701 | 26.44383 |
| B6 16-19 wks 2 A | mmu-miR-16-4373121 | 15.59256 |
| B6 16-19 wks 2 A | mmu-miR-17-4395419 | 17.16267 |
| B6 16-19 wks 2 A | mmu-miR-184-4373113 | 29.3225 |
| B6 16-19 wks 2 A | mmu-miR-186-4395396 | 22.24133 |
| B6 16-19 wks 2 A | mmu-miR-191-4395410 | 16.44813 |
| B6 16-19 wks 2 A | mmu-miR-192-4373108 | 19.97161 |
| B6 16-19 wks 2 A | mmu-miR-193b-4395597 | 20.95761 |
| B6 16-19 wks 2 A | mmu-miR-195-4373105 | 20.98666 |
| B6 16-19 wks 2 A | mmu-miR-199a-3p-4395415 | 24.75915 |
| B6 16-19 wks 2 A | mmu-miR-19a-4373099 | 19.65506 |
| B6 16-19 wks 2 A | mmu-miR-19b-4373098 | 15.61073 |
| B6 16-19 wks 2 A | mmu-miR-200b-4395362 | 23.38729 |
| B6 16-19 wks 2 A | mmu-miR-200c-4395411 | 25.01222 |
| B6 16-19 wks 2 A | mmu-miR-203-4373095 | 21.96495 |
| B6 16-19 wks 2 A | mmu-miR-204-4373094 | 26.40275 |
| B6 16-19 wks 2 A | mmu-miR-20a-4373286 | 17.91563 |
| B6 16-19 wks 2 A | mmu-miR-210-4373089 | 20.43666 |
| B6 16-19 wks 2 A | mmu-miR-21-4373090 | 17.56028 |
| B6 16-19 wks 2 A | mmu-miR-214-4395417 | 23.9689 |
| B6 16-19 wks 2 A | mmu-miR-215-4373316 | 20.95545 |
| B6 16-19 wks 2 A | mmu-miR-222-4395387 | 17.93006 |
| B6 16-19 wks 2 A | mmu-miR-223-4395406 | 17.05264 |
| B6 16-19 wks 2 A | mmu-miR-24-4373072 | 16.24518 |
| B6 16-19 wks 2 A | mmu-miR-25-4373071 | 20.22195 |
| B6 16-19 wks 2 A | mmu-miR-27a-4373287 | 22.0215 |
| B6 16-19 wks 2 A | mmu-miR-296-5p-4373066 | 23.59864 |
| **Sample** | **Detector** | ***C*_T_** |
| B6 16-19 wks 2 A | mmu-miR-29a-4395223 | 18.92536 |
| B6 16-19 wks 2 A | mmu-miR-29c-4395171 | 21.73187 |
| B6 16-19 wks 2 A | mmu-miR-30a-4373061 | 19.76288 |
| B6 16-19 wks 2 A | mmu-miR-30b-4373290 | 18.29378 |
| B6 16-19 wks 2 A | mmu-miR-30c-4373060 | 18.6414 |
| B6 16-19 wks 2 A | mmu-miR-30d-4373059 | 21.54289 |
| B6 16-19 wks 2 A | mmu-miR-30e-4395334 | 20.93902 |
| B6 16-19 wks 2 A | mmu-miR-320-4395388 | 21.19322 |
| B6 16-19 wks 2 A | mmu-miR-324-3p-4395639 | 23.21934 |
| B6 16-19 wks 2 A | mmu-miR-328-4373049 | 16.70241 |
| B6 16-19 wks 2 A | mmu-miR-331-3p-4373046 | 23.87791 |
| B6 16-19 wks 2 A | mmu-miR-339-3p-4395663 | 24.6659 |
| B6 16-19 wks 2 A | mmu-miR-342-3p-4395371 | 19.93987 |
| B6 16-19 wks 2 A | mmu-miR-365-4373194 | 23.73247 |
| B6 16-19 wks 2 A | mmu-miR-375-4373027 | 21.26358 |
| B6 16-19 wks 2 A | mmu-miR-409-3p-4395443 | 30.00116 |
| B6 16-19 wks 2 A | mmu-miR-434-3p-4395734 | 26.28918 |
| B6 16-19 wks 2 A | mmu-miR-451-4373360 | 16.97082 |
| B6 16-19 wks 2 A | mmu-miR-484-4381032 | 17.633 |
| B6 16-19 wks 2 A | mmu-miR-532-5p-4380928 | 22.68308 |
| B6 16-19 wks 2 A | mmu-miR-574-3p-4395460 | 20.76823 |
| B6 16-19 wks 2 A | mmu-miR-672-4395438 | 25.08672 |
| B6 16-19 wks 2 A | mmu-miR-676-4386776 | 23.99176 |
| B6 16-19 wks 2 A | mmu-miR-685-4386748 | 20.82597 |
| B6 16-19 wks 2 A | mmu-miR-744-4395435 | 22.95836 |
| B6 16-19 wks 2 A | mmu-miR-92a-4373013 | 17.3285 |
| B6 16-19 wks 2 A | mmu-miR-93-4373302 | 19.36288 |
| NOD 3-4 wks 1 A | MammU6-4395470 | 26.07592 |
| NOD 3-4 wks 1 A | MammU6-4395470 | 26.16353 |
| NOD 3-4 wks 1 A | MammU6-4395470 | 26.29417 |
| NOD 3-4 wks 1 A | MammU6-4395470 | 26.30084 |
| NOD 3-4 wks 1 A | mmu-let-7c-4373167 | 33.29952 |
| NOD 3-4 wks 1 A | mmu-let-7g-4395393 | 35.61163 |
| NOD 3-4 wks 1 A | mmu-miR-106a-4395589 | 32.40174 |
| NOD 3-4 wks 1 A | mmu-miR-125a-5p-4395309 | 35.94275 |
| NOD 3-4 wks 1 A | mmu-miR-125b-5p-4373148 | 35.93458 |
| NOD 3-4 wks 1 A | mmu-miR-126-3p-4395339 | 31.94867 |
| NOD 3-4 wks 1 A | mmu-miR-126-5p-4373269 | 34.95064 |
| NOD 3-4 wks 1 A | mmu-miR-133a-4395357 | 28.88973 |
| NOD 3-4 wks 1 A | mmu-miR-133b-4395358 | 33.14628 |
| NOD 3-4 wks 1 A | mmu-miR-134-4373299 | 33.58093 |
| NOD 3-4 wks 1 A | mmu-miR-139-5p-4395400 | 33.4724 |
| NOD 3-4 wks 1 A | mmu-miR-140-4373374 | 32.49706 |
| NOD 3-4 wks 1 A | mmu-miR-1-4395333 | 36.62709 |
| NOD 3-4 wks 1 A | mmu-miR-145-4395389 | 32.94456 |
| NOD 3-4 wks 1 A | mmu-miR-146a-4373132 | 32.1788 |
| NOD 3-4 wks 1 A | mmu-miR-146b-4373178 | 34.86422 |
| NOD 3-4 wks 1 A | mmu-miR-150-4373127 | 30.95388 |
| NOD 3-4 wks 1 A | mmu-miR-155-4395701 | 36.76361 |
| NOD 3-4 wks 1 A | mmu-miR-16-4373121 | 28.52617 |
| NOD 3-4 wks 1 A | mmu-miR-17-4395419 | 30.95428 |
| NOD 3-4 wks 1 A | mmu-miR-184-4373113 | 28.95917 |
| NOD 3-4 wks 1 A | mmu-miR-186-4395396 | 33.66857 |
| NOD 3-4 wks 1 A | mmu-miR-191-4395410 | 27.70084 |
| NOD 3-4 wks 1 A | mmu-miR-192-4373108 | 36.0078 |
| NOD 3-4 wks 1 A | mmu-miR-193b-4395597 | 31.93399 |
| NOD 3-4 wks 1 A | mmu-miR-195-4373105 | 35.00863 |
| NOD 3-4 wks 1 A | mmu-miR-199a-3p-4395415 | 35.18082 |
| NOD 3-4 wks 1 A | mmu-miR-19a-4373099 | 35.13284 |
| NOD 3-4 wks 1 A | mmu-miR-19b-4373098 | 30.67527 |
| NOD 3-4 wks 1 A | mmu-miR-200b-4395362 | 33.96523 |
| **Sample** | **Detector** | ***C*_T_** |
| NOD 3-4 wks 1 A | mmu-miR-200c-4395411 | 33.98886 |
| NOD 3-4 wks 1 A | mmu-miR-203-4373095 | 34.65333 |
| NOD 3-4 wks 1 A | mmu-miR-204-4373094 | 30.46473 |
| NOD 3-4 wks 1 A | mmu-miR-20a-4373286 | 32.85255 |
| NOD 3-4 wks 1 A | mmu-miR-210-4373089 | 33.3372 |
| NOD 3-4 wks 1 A | mmu-miR-21-4373090 | 33.50283 |
| NOD 3-4 wks 1 A | mmu-miR-214-4395417 | 35.97037 |
| NOD 3-4 wks 1 A | mmu-miR-215-4373316 | 36.9275 |
| NOD 3-4 wks 1 A | mmu-miR-222-4395387 | 28.50472 |
| NOD 3-4 wks 1 A | mmu-miR-223-4395406 | 27.93887 |
| NOD 3-4 wks 1 A | mmu-miR-24-4373072 | 28.75638 |
| NOD 3-4 wks 1 A | mmu-miR-25-4373071 | 34.45992 |
| NOD 3-4 wks 1 A | mmu-miR-27a-4373287 | 35.47248 |
| NOD 3-4 wks 1 A | mmu-miR-296-5p-4373066 | 34.9788 |
| NOD 3-4 wks 1 A | mmu-miR-29a-4395223 | 32.78478 |
| NOD 3-4 wks 1 A | mmu-miR-29c-4395171 | 37.01574 |
| NOD 3-4 wks 1 A | mmu-miR-30a-4373061 | 33.32225 |
| NOD 3-4 wks 1 A | mmu-miR-30b-4373290 | 33.61395 |
| NOD 3-4 wks 1 A | mmu-miR-30c-4373060 | 32.97746 |
| NOD 3-4 wks 1 A | mmu-miR-30d-4373059 | 33.50319 |
| NOD 3-4 wks 1 A | mmu-miR-30e-4395334 | 33.93176 |
| NOD 3-4 wks 1 A | mmu-miR-320-4395388 | 31.95559 |
| NOD 3-4 wks 1 A | mmu-miR-324-3p-4395639 | 34.89321 |
| NOD 3-4 wks 1 A | mmu-miR-328-4373049 | 30.93359 |
| NOD 3-4 wks 1 A | mmu-miR-331-3p-4373046 | 36.94657 |
| NOD 3-4 wks 1 A | mmu-miR-339-3p-4395663 | 32.94988 |
| NOD 3-4 wks 1 A | mmu-miR-342-3p-4395371 | 34.27951 |
| NOD 3-4 wks 1 A | mmu-miR-365-4373194 | 35.87315 |
| NOD 3-4 wks 1 A | mmu-miR-375-4373027 | 36.93973 |
| NOD 3-4 wks 1 A | mmu-miR-409-3p-4395443 | 34.59761 |
| NOD 3-4 wks 1 A | mmu-miR-434-3p-4395734 | 35.94335 |
| NOD 3-4 wks 1 A | mmu-miR-451-4373360 | 34.35226 |
| NOD 3-4 wks 1 A | mmu-miR-484-4381032 | 27.5209 |
| NOD 3-4 wks 1 A | mmu-miR-532-5p-4380928 | 36.01609 |
| NOD 3-4 wks 1 A | mmu-miR-574-3p-4395460 | 30.97931 |
| NOD 3-4 wks 1 A | mmu-miR-672-4395438 | 35.95304 |
| NOD 3-4 wks 1 A | mmu-miR-676-4386776 | 33.69548 |
| NOD 3-4 wks 1 A | mmu-miR-685-4386748 | 30.67827 |
| NOD 3-4 wks 1 A | mmu-miR-744-4395435 | 36.93998 |
| NOD 3-4 wks 1 A | mmu-miR-92a-4373013 | 31.22715 |
| NOD 3-4 wks 1 A | mmu-miR-93-4373302 | 32.87117 |
| NOD 3-4 wks 2 A | MammU6-4395470 | 20.70802 |
| NOD 3-4 wks 2 A | MammU6-4395470 | 20.72198 |
| NOD 3-4 wks 2 A | MammU6-4395470 | 20.93729 |
| NOD 3-4 wks 2 A | MammU6-4395470 | 20.98625 |
| NOD 3-4 wks 2 A | mmu-let-7c-4373167 | 23.829 |
| NOD 3-4 wks 2 A | mmu-let-7g-4395393 | 25.15864 |
| NOD 3-4 wks 2 A | mmu-miR-106a-4395589 | 21.69168 |
| NOD 3-4 wks 2 A | mmu-miR-125a-5p-4395309 | 25.66288 |
| NOD 3-4 wks 2 A | mmu-miR-125b-5p-4373148 | 25.05646 |
| NOD 3-4 wks 2 A | mmu-miR-126-3p-4395339 | 22.68382 |
| NOD 3-4 wks 2 A | mmu-miR-126-5p-4373269 | 24.18436 |
| NOD 3-4 wks 2 A | mmu-miR-133a-4395357 | 18.97479 |
| NOD 3-4 wks 2 A | mmu-miR-133b-4395358 | 23.50672 |
| NOD 3-4 wks 2 A | mmu-miR-134-4373299 | 25.93809 |
| NOD 3-4 wks 2 A | mmu-miR-139-5p-4395400 | 24.18579 |
| NOD 3-4 wks 2 A | mmu-miR-140-4373374 | 22.95252 |
| NOD 3-4 wks 2 A | mmu-miR-1-4395333 | 25.14654 |
| NOD 3-4 wks 2 A | mmu-miR-145-4395389 | 21.98777 |
| NOD 3-4 wks 2 A | mmu-miR-146a-4373132 | 23.71154 |
| NOD 3-4 wks 2 A | mmu-miR-146b-4373178 | 26.76961 |
| **Sample** | **Detector** | ***C*_T_** |
| NOD 3-4 wks 2 A | mmu-miR-150-4373127 | 20.46642 |
| NOD 3-4 wks 2 A | mmu-miR-155-4395701 | 28.88226 |
| NOD 3-4 wks 2 A | mmu-miR-16-4373121 | 18.95505 |
| NOD 3-4 wks 2 A | mmu-miR-17-4395419 | 21.6665 |
| NOD 3-4 wks 2 A | mmu-miR-184-4373113 | 29.9681 |
| NOD 3-4 wks 2 A | mmu-miR-186-4395396 | 23.6998 |
| NOD 3-4 wks 2 A | mmu-miR-191-4395410 | 19.45559 |
| NOD 3-4 wks 2 A | mmu-miR-192-4373108 | 26.01727 |
| NOD 3-4 wks 2 A | mmu-miR-193b-4395597 | 23.03593 |
| NOD 3-4 wks 2 A | mmu-miR-195-4373105 | 23.75453 |
| NOD 3-4 wks 2 A | mmu-miR-199a-3p-4395415 | 27.54342 |
| NOD 3-4 wks 2 A | mmu-miR-19a-4373099 | 24.58732 |
| NOD 3-4 wks 2 A | mmu-miR-19b-4373098 | 20.75176 |
| NOD 3-4 wks 2 A | mmu-miR-200b-4395362 | 26.99901 |
| NOD 3-4 wks 2 A | mmu-miR-200c-4395411 | 27.7083 |
| NOD 3-4 wks 2 A | mmu-miR-203-4373095 | 26.97038 |
| NOD 3-4 wks 2 A | mmu-miR-204-4373094 | 27.9639 |
| NOD 3-4 wks 2 A | mmu-miR-20a-4373286 | 21.67765 |
| NOD 3-4 wks 2 A | mmu-miR-210-4373089 | 23.61749 |
| NOD 3-4 wks 2 A | mmu-miR-21-4373090 | 23.99878 |
| NOD 3-4 wks 2 A | mmu-miR-214-4395417 | 26.30412 |
| NOD 3-4 wks 2 A | mmu-miR-215-4373316 | 27.122 |
| NOD 3-4 wks 2 A | mmu-miR-222-4395387 | 19.93927 |
| NOD 3-4 wks 2 A | mmu-miR-223-4395406 | 20.21194 |
| NOD 3-4 wks 2 A | mmu-miR-24-4373072 | 19.09419 |
| NOD 3-4 wks 2 A | mmu-miR-25-4373071 | 24.54988 |
| NOD 3-4 wks 2 A | mmu-miR-27a-4373287 | 26.93846 |
| NOD 3-4 wks 2 A | mmu-miR-296-5p-4373066 | 22.59251 |
| NOD 3-4 wks 2 A | mmu-miR-29a-4395223 | 23.88641 |
| NOD 3-4 wks 2 A | mmu-miR-29c-4395171 | 24.11166 |
| NOD 3-4 wks 2 A | mmu-miR-30a-4373061 | 23.65089 |
| NOD 3-4 wks 2 A | mmu-miR-30b-4373290 | 22.56247 |
| NOD 3-4 wks 2 A | mmu-miR-30c-4373060 | 22.44212 |
| NOD 3-4 wks 2 A | mmu-miR-30d-4373059 | 24.97579 |
| NOD 3-4 wks 2 A | mmu-miR-30e-4395334 | 24.16702 |
| NOD 3-4 wks 2 A | mmu-miR-320-4395388 | 23.75872 |
| NOD 3-4 wks 2 A | mmu-miR-324-3p-4395639 | 25.34189 |
| NOD 3-4 wks 2 A | mmu-miR-328-4373049 | 19.55261 |
| NOD 3-4 wks 2 A | mmu-miR-331-3p-4373046 | 25.52165 |
| NOD 3-4 wks 2 A | mmu-miR-339-3p-4395663 | 24.94818 |
| NOD 3-4 wks 2 A | mmu-miR-342-3p-4395371 | 24.50547 |
| NOD 3-4 wks 2 A | mmu-miR-365-4373194 | 27.04162 |
| NOD 3-4 wks 2 A | mmu-miR-375-4373027 | 23.48641 |
| NOD 3-4 wks 2 A | mmu-miR-409-3p-4395443 | 23.99373 |
| NOD 3-4 wks 2 A | mmu-miR-434-3p-4395734 | 25.9741 |
| NOD 3-4 wks 2 A | mmu-miR-451-4373360 | 23.57772 |
| NOD 3-4 wks 2 A | mmu-miR-484-4381032 | 19.2826 |
| NOD 3-4 wks 2 A | mmu-miR-532-5p-4380928 | 24.82171 |
| NOD 3-4 wks 2 A | mmu-miR-574-3p-4395460 | 22.86054 |
| NOD 3-4 wks 2 A | mmu-miR-672-4395438 | 27.2708 |
| NOD 3-4 wks 2 A | mmu-miR-676-4386776 | 25.04064 |
| NOD 3-4 wks 2 A | mmu-miR-685-4386748 | 22.57705 |
| NOD 3-4 wks 2 A | mmu-miR-744-4395435 | 25.11472 |
| NOD 3-4 wks 2 A | mmu-miR-92a-4373013 | 18.94293 |
| NOD 3-4 wks 2 A | mmu-miR-93-4373302 | 23.44723 |
| NOD 7-8 wks 1 A | MammU6-4395470 | 28.58521 |
| NOD 7-8 wks 1 A | MammU6-4395470 | 28.95672 |
| NOD 7-8 wks 1 A | MammU6-4395470 | 29.04041 |
| NOD 7-8 wks 1 A | MammU6-4395470 | 29.14849 |
| NOD 7-8 wks 1 A | mmu-let-7c-4373167 | 28.325 |
| NOD 7-8 wks 1 A | mmu-let-7g-4395393 | 30.18553 |
| **Sample** | **Detector** | ***C*_T_** |
| NOD 7-8 wks 1 A | mmu-miR-106a-4395589 | 26.09006 |
| NOD 7-8 wks 1 A | mmu-miR-125a-5p-4395309 | 30.87395 |
| NOD 7-8 wks 1 A | mmu-miR-125b-5p-4373148 | 29.49226 |
| NOD 7-8 wks 1 A | mmu-miR-126-3p-4395339 | 27.9907 |
| NOD 7-8 wks 1 A | mmu-miR-126-5p-4373269 | 31.79968 |
| NOD 7-8 wks 1 A | mmu-miR-133a-4395357 | 24.31422 |
| NOD 7-8 wks 1 A | mmu-miR-133b-4395358 | 28.97658 |
| NOD 7-8 wks 1 A | mmu-miR-134-4373299 | 28.98983 |
| NOD 7-8 wks 1 A | mmu-miR-139-5p-4395400 | 28.95058 |
| NOD 7-8 wks 1 A | mmu-miR-140-4373374 | 26.67723 |
| NOD 7-8 wks 1 A | mmu-miR-1-4395333 | 31.08611 |
| NOD 7-8 wks 1 A | mmu-miR-145-4395389 | 23.69163 |
| NOD 7-8 wks 1 A | mmu-miR-146a-4373132 | 26.26134 |
| NOD 7-8 wks 1 A | mmu-miR-146b-4373178 | 28.76394 |
| NOD 7-8 wks 1 A | mmu-miR-150-4373127 | 24.5226 |
| NOD 7-8 wks 1 A | mmu-miR-155-4395701 | 32.64283 |
| NOD 7-8 wks 1 A | mmu-miR-16-4373121 | 24.64098 |
| NOD 7-8 wks 1 A | mmu-miR-17-4395419 | 26.15856 |
| NOD 7-8 wks 1 A | mmu-miR-184-4373113 | 26.27006 |
| NOD 7-8 wks 1 A | mmu-miR-186-4395396 | 28.02788 |
| NOD 7-8 wks 1 A | mmu-miR-191-4395410 | 22.63027 |
| NOD 7-8 wks 1 A | mmu-miR-192-4373108 | 29.14938 |
| NOD 7-8 wks 1 A | mmu-miR-193b-4395597 | 26.89437 |
| NOD 7-8 wks 1 A | mmu-miR-195-4373105 | 30.65417 |
| NOD 7-8 wks 1 A | mmu-miR-199a-3p-4395415 | 30.54357 |
| NOD 7-8 wks 1 A | mmu-miR-19a-4373099 | 29.93729 |
| NOD 7-8 wks 1 A | mmu-miR-19b-4373098 | 24.51017 |
| NOD 7-8 wks 1 A | mmu-miR-200b-4395362 | 29.02686 |
| NOD 7-8 wks 1 A | mmu-miR-200c-4395411 | 31.17324 |
| NOD 7-8 wks 1 A | mmu-miR-203-4373095 | 31.29755 |
| NOD 7-8 wks 1 A | mmu-miR-204-4373094 | 28.27552 |
| NOD 7-8 wks 1 A | mmu-miR-20a-4373286 | 28.18294 |
| NOD 7-8 wks 1 A | mmu-miR-210-4373089 | 29.42544 |
| NOD 7-8 wks 1 A | mmu-miR-21-4373090 | 28.17052 |
| NOD 7-8 wks 1 A | mmu-miR-214-4395417 | 29.74877 |
| NOD 7-8 wks 1 A | mmu-miR-215-4373316 | 31.02957 |
| NOD 7-8 wks 1 A | mmu-miR-222-4395387 | 23.42943 |
| NOD 7-8 wks 1 A | mmu-miR-223-4395406 | 22.40482 |
| NOD 7-8 wks 1 A | mmu-miR-24-4373072 | 23.69277 |
| NOD 7-8 wks 1 A | mmu-miR-25-4373071 | 28.58031 |
| NOD 7-8 wks 1 A | mmu-miR-27a-4373287 | 34.95772 |
| NOD 7-8 wks 1 A | mmu-miR-296-5p-4373066 | 28.3674 |
| NOD 7-8 wks 1 A | mmu-miR-29a-4395223 | 29.64046 |
| NOD 7-8 wks 1 A | mmu-miR-29c-4395171 | 31.82233 |
| NOD 7-8 wks 1 A | mmu-miR-30a-4373061 | 28.78751 |
| NOD 7-8 wks 1 A | mmu-miR-30b-4373290 | 26.90061 |
| NOD 7-8 wks 1 A | mmu-miR-30c-4373060 | 26.86511 |
| NOD 7-8 wks 1 A | mmu-miR-30d-4373059 | 30.53426 |
| NOD 7-8 wks 1 A | mmu-miR-30e-4395334 | 29.38111 |
| NOD 7-8 wks 1 A | mmu-miR-320-4395388 | 27.86719 |
| NOD 7-8 wks 1 A | mmu-miR-324-3p-4395639 | 29.95098 |
| NOD 7-8 wks 1 A | mmu-miR-328-4373049 | 22.4957 |
| NOD 7-8 wks 1 A | mmu-miR-331-3p-4373046 | 31.10852 |
| NOD 7-8 wks 1 A | mmu-miR-339-3p-4395663 | 29.71514 |
| NOD 7-8 wks 1 A | mmu-miR-342-3p-4395371 | 27.82908 |
| NOD 7-8 wks 1 A | mmu-miR-365-4373194 | 29.95736 |
| NOD 7-8 wks 1 A | mmu-miR-375-4373027 | 27.67803 |
| NOD 7-8 wks 1 A | mmu-miR-409-3p-4395443 | 27.0438 |
| NOD 7-8 wks 1 A | mmu-miR-434-3p-4395734 | 29.38049 |
| NOD 7-8 wks 1 A | mmu-miR-451-4373360 | 27.47527 |
| NOD 7-8 wks 1 A | mmu-miR-484-4381032 | 21.66864 |
| **Sample** | **Detector** | ***C*_T_** |
| NOD 7-8 wks 1 A | mmu-miR-532-5p-4380928 | 27.811 |
| NOD 7-8 wks 1 A | mmu-miR-574-3p-4395460 | 26.69815 |
| NOD 7-8 wks 1 A | mmu-miR-672-4395438 | 28.50002 |
| NOD 7-8 wks 1 A | mmu-miR-676-4386776 | 28.2071 |
| NOD 7-8 wks 1 A | mmu-miR-685-4386748 | 28.97833 |
| NOD 7-8 wks 1 A | mmu-miR-744-4395435 | 30.02379 |
| NOD 7-8 wks 1 A | mmu-miR-92a-4373013 | 23.49809 |
| NOD 7-8 wks 1 A | mmu-miR-93-4373302 | 27.40554 |
| NOD 7-8 wks 2 A | MammU6-4395470 | 20.70536 |
| NOD 7-8 wks 2 A | MammU6-4395470 | 20.90293 |
| NOD 7-8 wks 2 A | MammU6-4395470 | 20.91626 |
| NOD 7-8 wks 2 A | MammU6-4395470 | 20.9814 |
| NOD 7-8 wks 2 A | mmu-let-7c-4373167 | 24.44664 |
| NOD 7-8 wks 2 A | mmu-let-7g-4395393 | 26.33732 |
| NOD 7-8 wks 2 A | mmu-miR-106a-4395589 | 21.97374 |
| NOD 7-8 wks 2 A | mmu-miR-125a-5p-4395309 | 24.28936 |
| NOD 7-8 wks 2 A | mmu-miR-125b-5p-4373148 | 25.25713 |
| NOD 7-8 wks 2 A | mmu-miR-126-3p-4395339 | 23.06699 |
| NOD 7-8 wks 2 A | mmu-miR-126-5p-4373269 | 24.95653 |
| NOD 7-8 wks 2 A | mmu-miR-133a-4395357 | 19.74431 |
| NOD 7-8 wks 2 A | mmu-miR-133b-4395358 | 24.63107 |
| NOD 7-8 wks 2 A | mmu-miR-134-4373299 | 26.32204 |
| NOD 7-8 wks 2 A | mmu-miR-139-5p-4395400 | 23.51187 |
| NOD 7-8 wks 2 A | mmu-miR-140-4373374 | 24.46576 |
| NOD 7-8 wks 2 A | mmu-miR-1-4395333 | 26.28576 |
| NOD 7-8 wks 2 A | mmu-miR-145-4395389 | 20.84428 |
| NOD 7-8 wks 2 A | mmu-miR-146a-4373132 | 23.47989 |
| NOD 7-8 wks 2 A | mmu-miR-146b-4373178 | 26.86114 |
| NOD 7-8 wks 2 A | mmu-miR-150-4373127 | 19.54814 |
| NOD 7-8 wks 2 A | mmu-miR-155-4395701 | 28.81185 |
| NOD 7-8 wks 2 A | mmu-miR-16-4373121 | 18.61372 |
| NOD 7-8 wks 2 A | mmu-miR-17-4395419 | 21.93634 |
| NOD 7-8 wks 2 A | mmu-miR-184-4373113 | 23.74152 |
| NOD 7-8 wks 2 A | mmu-miR-186-4395396 | 22.94496 |
| NOD 7-8 wks 2 A | mmu-miR-191-4395410 | 18.69192 |
| NOD 7-8 wks 2 A | mmu-miR-192-4373108 | 25.19828 |
| NOD 7-8 wks 2 A | mmu-miR-193b-4395597 | 22.33174 |
| NOD 7-8 wks 2 A | mmu-miR-195-4373105 | 23.48223 |
| NOD 7-8 wks 2 A | mmu-miR-199a-3p-4395415 | 27.68999 |
| NOD 7-8 wks 2 A | mmu-miR-19a-4373099 | 25.6266 |
| NOD 7-8 wks 2 A | mmu-miR-19b-4373098 | 21.75167 |
| NOD 7-8 wks 2 A | mmu-miR-200b-4395362 | 25.55608 |
| NOD 7-8 wks 2 A | mmu-miR-200c-4395411 | 27.093 |
| NOD 7-8 wks 2 A | mmu-miR-203-4373095 | 25.81016 |
| NOD 7-8 wks 2 A | mmu-miR-204-4373094 | 24.47969 |
| NOD 7-8 wks 2 A | mmu-miR-20a-4373286 | 22.7933 |
| NOD 7-8 wks 2 A | mmu-miR-210-4373089 | 24.71225 |
| NOD 7-8 wks 2 A | mmu-miR-21-4373090 | 24.91474 |
| NOD 7-8 wks 2 A | mmu-miR-214-4395417 | 25.10843 |
| NOD 7-8 wks 2 A | mmu-miR-215-4373316 | 26.86487 |
| NOD 7-8 wks 2 A | mmu-miR-222-4395387 | 20.15645 |
| NOD 7-8 wks 2 A | mmu-miR-223-4395406 | 19.81185 |
| NOD 7-8 wks 2 A | mmu-miR-24-4373072 | 19.63476 |
| NOD 7-8 wks 2 A | mmu-miR-25-4373071 | 24.93663 |
| NOD 7-8 wks 2 A | mmu-miR-27a-4373287 | 28.0615 |
| NOD 7-8 wks 2 A | mmu-miR-296-5p-4373066 | 24.23582 |
| NOD 7-8 wks 2 A | mmu-miR-29a-4395223 | 23.99278 |
| NOD 7-8 wks 2 A | mmu-miR-29c-4395171 | 24.38372 |
| NOD 7-8 wks 2 A | mmu-miR-30a-4373061 | 24.19755 |
| NOD 7-8 wks 2 A | mmu-miR-30b-4373290 | 23.46893 |
| NOD 7-8 wks 2 A | mmu-miR-30c-4373060 | 22.91716 |
| **Sample** | **Detector** | ***C*_T_** |
| NOD 7-8 wks 2 A | mmu-miR-30d-4373059 | 25.60236 |
| NOD 7-8 wks 2 A | mmu-miR-30e-4395334 | 24.22715 |
| NOD 7-8 wks 2 A | mmu-miR-320-4395388 | 22.81751 |
| NOD 7-8 wks 2 A | mmu-miR-324-3p-4395639 | 24.83449 |
| NOD 7-8 wks 2 A | mmu-miR-328-4373049 | 19.3377 |
| NOD 7-8 wks 2 A | mmu-miR-331-3p-4373046 | 25.03493 |
| NOD 7-8 wks 2 A | mmu-miR-339-3p-4395663 | 25.20242 |
| NOD 7-8 wks 2 A | mmu-miR-342-3p-4395371 | 24.03849 |
| NOD 7-8 wks 2 A | mmu-miR-365-4373194 | 26.76746 |
| NOD 7-8 wks 2 A | mmu-miR-375-4373027 | 23.99884 |
| NOD 7-8 wks 2 A | mmu-miR-409-3p-4395443 | 22.96981 |
| NOD 7-8 wks 2 A | mmu-miR-434-3p-4395734 | 25.04975 |
| NOD 7-8 wks 2 A | mmu-miR-451-4373360 | 23.45208 |
| NOD 7-8 wks 2 A | mmu-miR-484-4381032 | 18.94557 |
| NOD 7-8 wks 2 A | mmu-miR-532-5p-4380928 | 26.02371 |
| NOD 7-8 wks 2 A | mmu-miR-574-3p-4395460 | 22.3323 |
| NOD 7-8 wks 2 A | mmu-miR-672-4395438 | 26.07889 |
| NOD 7-8 wks 2 A | mmu-miR-676-4386776 | 24.94283 |
| NOD 7-8 wks 2 A | mmu-miR-685-4386748 | 23.32231 |
| NOD 7-8 wks 2 A | mmu-miR-744-4395435 | 25.65506 |
| NOD 7-8 wks 2 A | mmu-miR-92a-4373013 | 18.4074 |
| NOD 7-8 wks 2 A | mmu-miR-93-4373302 | 24.61745 |
| NOD 16-19 wks 1 A | MammU6-4395470 | 27.78407 |
| NOD 16-19 wks 1 A | MammU6-4395470 | 27.86787 |
| NOD 16-19 wks 1 A | MammU6-4395470 | 27.97779 |
| NOD 16-19 wks 1 A | MammU6-4395470 | 28.15551 |
| NOD 16-19 wks 1 A | mmu-let-7c-4373167 | 29.87201 |
| NOD 16-19 wks 1 A | mmu-let-7g-4395393 | 31.9557 |
| NOD 16-19 wks 1 A | mmu-miR-106a-4395589 | 25.37025 |
| NOD 16-19 wks 1 A | mmu-miR-125a-5p-4395309 | 31.56047 |
| NOD 16-19 wks 1 A | mmu-miR-125b-5p-4373148 | 30.96453 |
| NOD 16-19 wks 1 A | mmu-miR-126-3p-4395339 | 26.78167 |
| NOD 16-19 wks 1 A | mmu-miR-126-5p-4373269 | 29.89064 |
| NOD 16-19 wks 1 A | mmu-miR-133a-4395357 | 23.57546 |
| NOD 16-19 wks 1 A | mmu-miR-133b-4395358 | 28.23358 |
| NOD 16-19 wks 1 A | mmu-miR-134-4373299 | 27.73963 |
| NOD 16-19 wks 1 A | mmu-miR-139-5p-4395400 | 27.54536 |
| NOD 16-19 wks 1 A | mmu-miR-140-4373374 | 26.38029 |
| NOD 16-19 wks 1 A | mmu-miR-1-4395333 | 34.03876 |
| NOD 16-19 wks 1 A | mmu-miR-145-4395389 | 24.16609 |
| NOD 16-19 wks 1 A | mmu-miR-146a-4373132 | 25.0014 |
| NOD 16-19 wks 1 A | mmu-miR-146b-4373178 | 27.84905 |
| NOD 16-19 wks 1 A | mmu-miR-150-4373127 | 23.37853 |
| NOD 16-19 wks 1 A | mmu-miR-155-4395701 | 32.39376 |
| NOD 16-19 wks 1 A | mmu-miR-16-4373121 | 23.3532 |
| NOD 16-19 wks 1 A | mmu-miR-17-4395419 | 25.34378 |
| NOD 16-19 wks 1 A | mmu-miR-184-4373113 | 28.08328 |
| NOD 16-19 wks 1 A | mmu-miR-186-4395396 | 27.37349 |
| NOD 16-19 wks 1 A | mmu-miR-191-4395410 | 21.59154 |
| NOD 16-19 wks 1 A | mmu-miR-192-4373108 | 28.87881 |
| NOD 16-19 wks 1 A | mmu-miR-193b-4395597 | 25.25173 |
| NOD 16-19 wks 1 A | mmu-miR-195-4373105 | 29.40911 |
| NOD 16-19 wks 1 A | mmu-miR-199a-3p-4395415 | 30.5195 |
| NOD 16-19 wks 1 A | mmu-miR-19a-4373099 | 27.9307 |
| NOD 16-19 wks 1 A | mmu-miR-19b-4373098 | 23.47785 |
| NOD 16-19 wks 1 A | mmu-miR-200b-4395362 | 28.25566 |
| NOD 16-19 wks 1 A | mmu-miR-200c-4395411 | 31.18243 |
| NOD 16-19 wks 1 A | mmu-miR-203-4373095 | 28.55076 |
| NOD 16-19 wks 1 A | mmu-miR-204-4373094 | 29.82975 |
| NOD 16-19 wks 1 A | mmu-miR-20a-4373286 | 27.48998 |
| NOD 16-19 wks 1 A | mmu-miR-210-4373089 | 28.07375 |
| **Sample** | **Detector** | ***C*_T_** |
| NOD 16-19 wks 1 A | mmu-miR-21-4373090 | 27.95284 |
| NOD 16-19 wks 1 A | mmu-miR-214-4395417 | 30.92302 |
| NOD 16-19 wks 1 A | mmu-miR-215-4373316 | 29.87303 |
| NOD 16-19 wks 1 A | mmu-miR-222-4395387 | 22.16687 |
| NOD 16-19 wks 1 A | mmu-miR-223-4395406 | 21.18111 |
| NOD 16-19 wks 1 A | mmu-miR-24-4373072 | 22.20861 |
| NOD 16-19 wks 1 A | mmu-miR-25-4373071 | 28.55059 |
| NOD 16-19 wks 1 A | mmu-miR-27a-4373287 | 30.56997 |
| NOD 16-19 wks 1 A | mmu-miR-296-5p-4373066 | 29.48893 |
| NOD 16-19 wks 1 A | mmu-miR-29a-4395223 | 27.91727 |
| NOD 16-19 wks 1 A | mmu-miR-29c-4395171 | 30.88229 |
| NOD 16-19 wks 1 A | mmu-miR-30a-4373061 | 28.94907 |
| NOD 16-19 wks 1 A | mmu-miR-30b-4373290 | 26.97178 |
| NOD 16-19 wks 1 A | mmu-miR-30c-4373060 | 27.53881 |
| NOD 16-19 wks 1 A | mmu-miR-30d-4373059 | 30.95968 |
| NOD 16-19 wks 1 A | mmu-miR-30e-4395334 | 28.86586 |
| NOD 16-19 wks 1 A | mmu-miR-320-4395388 | 27.3544 |
| NOD 16-19 wks 1 A | mmu-miR-324-3p-4395639 | 28.29576 |
| NOD 16-19 wks 1 A | mmu-miR-328-4373049 | 21.97556 |
| NOD 16-19 wks 1 A | mmu-miR-331-3p-4373046 | 30.35475 |
| NOD 16-19 wks 1 A | mmu-miR-339-3p-4395663 | 26.72023 |
| NOD 16-19 wks 1 A | mmu-miR-342-3p-4395371 | 26.26808 |
| NOD 16-19 wks 1 A | mmu-miR-365-4373194 | 29.74959 |
| NOD 16-19 wks 1 A | mmu-miR-375-4373027 | 27.05247 |
| NOD 16-19 wks 1 A | mmu-miR-409-3p-4395443 | 26.06042 |
| NOD 16-19 wks 1 A | mmu-miR-434-3p-4395734 | 28.71682 |
| NOD 16-19 wks 1 A | mmu-miR-451-4373360 | 28.97452 |
| NOD 16-19 wks 1 A | mmu-miR-484-4381032 | 20.21804 |
| NOD 16-19 wks 1 A | mmu-miR-532-5p-4380928 | 26.36928 |
| NOD 16-19 wks 1 A | mmu-miR-574-3p-4395460 | 25.67694 |
| NOD 16-19 wks 1 A | mmu-miR-672-4395438 | 28.95447 |
| NOD 16-19 wks 1 A | mmu-miR-676-4386776 | 27.85522 |
| NOD 16-19 wks 1 A | mmu-miR-685-4386748 | 26.33872 |
| NOD 16-19 wks 1 A | mmu-miR-744-4395435 | 29.5843 |
| NOD 16-19 wks 1 A | mmu-miR-92a-4373013 | 23.5499 |
| NOD 16-19 wks 1 A | mmu-miR-93-4373302 | 27.69834 |
| NOD 16-19 wks 2 A | MammU6-4395470 | 20.08048 |
| NOD 16-19 wks 2 A | MammU6-4395470 | 20.30009 |
| NOD 16-19 wks 2 A | MammU6-4395470 | 20.30732 |
| NOD 16-19 wks 2 A | MammU6-4395470 | 20.78141 |
| NOD 16-19 wks 2 A | mmu-let-7c-4373167 | 28.0164 |
| NOD 16-19 wks 2 A | mmu-let-7g-4395393 | 28.99522 |
| NOD 16-19 wks 2 A | mmu-miR-106a-4395589 | 22.98341 |
| NOD 16-19 wks 2 A | mmu-miR-125a-5p-4395309 | 26.49 |
| NOD 16-19 wks 2 A | mmu-miR-125b-5p-4373148 | 29.09874 |
| NOD 16-19 wks 2 A | mmu-miR-126-3p-4395339 | 23.34792 |
| NOD 16-19 wks 2 A | mmu-miR-126-5p-4373269 | 26.27587 |
| NOD 16-19 wks 2 A | mmu-miR-133a-4395357 | 19.30179 |
| NOD 16-19 wks 2 A | mmu-miR-133b-4395358 | 27.07011 |
| NOD 16-19 wks 2 A | mmu-miR-134-4373299 | 25.661 |
| NOD 16-19 wks 2 A | mmu-miR-139-5p-4395400 | 24.98159 |
| NOD 16-19 wks 2 A | mmu-miR-140-4373374 | 26.23605 |
| NOD 16-19 wks 2 A | mmu-miR-1-4395333 | 29.95427 |
| NOD 16-19 wks 2 A | mmu-miR-145-4395389 | 24.28442 |
| NOD 16-19 wks 2 A | mmu-miR-146a-4373132 | 21.96637 |
| NOD 16-19 wks 2 A | mmu-miR-146b-4373178 | 23.93075 |
| NOD 16-19 wks 2 A | mmu-miR-150-4373127 | 18.31851 |
| NOD 16-19 wks 2 A | mmu-miR-155-4395701 | 27.2368 |
| NOD 16-19 wks 2 A | mmu-miR-16-4373121 | 18.73616 |
| NOD 16-19 wks 2 A | mmu-miR-17-4395419 | 23.40426 |
| NOD 16-19 wks 2 A | mmu-miR-184-4373113 | 24.50512 |
| **Sample** | **Detector** | ***C*_T_** |
| NOD 16-19 wks 2 A | mmu-miR-186-4395396 | 22.95968 |
| NOD 16-19 wks 2 A | mmu-miR-191-4395410 | 16.93768 |
| NOD 16-19 wks 2 A | mmu-miR-192-4373108 | 25.44702 |
| NOD 16-19 wks 2 A | mmu-miR-193b-4395597 | 23.15842 |
| NOD 16-19 wks 2 A | mmu-miR-195-4373105 | 24.4256 |
| NOD 16-19 wks 2 A | mmu-miR-199a-3p-4395415 | 29.98623 |
| NOD 16-19 wks 2 A | mmu-miR-19a-4373099 | 27.60088 |
| NOD 16-19 wks 2 A | mmu-miR-19b-4373098 | 23.96945 |
| NOD 16-19 wks 2 A | mmu-miR-200b-4395362 | 27.34603 |
| NOD 16-19 wks 2 A | mmu-miR-200c-4395411 | 27.50623 |
| NOD 16-19 wks 2 A | mmu-miR-203-4373095 | 25.39894 |
| NOD 16-19 wks 2 A | mmu-miR-204-4373094 | 26.59953 |
| NOD 16-19 wks 2 A | mmu-miR-20a-4373286 | 25.97826 |
| NOD 16-19 wks 2 A | mmu-miR-210-4373089 | 25.17804 |
| NOD 16-19 wks 2 A | mmu-miR-21-4373090 | 26.86149 |
| NOD 16-19 wks 2 A | mmu-miR-214-4395417 | 25.67312 |
| NOD 16-19 wks 2 A | mmu-miR-215-4373316 | 27.24593 |
| NOD 16-19 wks 2 A | mmu-miR-222-4395387 | 20.48035 |
| NOD 16-19 wks 2 A | mmu-miR-223-4395406 | 19.69404 |
| NOD 16-19 wks 2 A | mmu-miR-24-4373072 | 19.0764 |
| NOD 16-19 wks 2 A | mmu-miR-25-4373071 | 28.51914 |
| NOD 16-19 wks 2 A | mmu-miR-27a-4373287 | 29.06398 |
| NOD 16-19 wks 2 A | mmu-miR-296-5p-4373066 | 32.49224 |
| NOD 16-19 wks 2 A | mmu-miR-29a-4395223 | 23.9356 |
| NOD 16-19 wks 2 A | mmu-miR-29c-4395171 | 24.75103 |
| NOD 16-19 wks 2 A | mmu-miR-30a-4373061 | 26.40307 |
| NOD 16-19 wks 2 A | mmu-miR-30b-4373290 | 26.62323 |
| NOD 16-19 wks 2 A | mmu-miR-30c-4373060 | 26.9825 |
| NOD 16-19 wks 2 A | mmu-miR-30d-4373059 | 27.83157 |
| NOD 16-19 wks 2 A | mmu-miR-30e-4395334 | 24.2094 |
| NOD 16-19 wks 2 A | mmu-miR-320-4395388 | 22.23732 |
| NOD 16-19 wks 2 A | mmu-miR-324-3p-4395639 | 25.89018 |
| NOD 16-19 wks 2 A | mmu-miR-328-4373049 | 23.80063 |
| NOD 16-19 wks 2 A | mmu-miR-331-3p-4373046 | 27.76141 |
| NOD 16-19 wks 2 A | mmu-miR-339-3p-4395663 | 22.2472 |
| NOD 16-19 wks 2 A | mmu-miR-342-3p-4395371 | 22.96987 |
| NOD 16-19 wks 2 A | mmu-miR-365-4373194 | 30.24709 |
| NOD 16-19 wks 2 A | mmu-miR-375-4373027 | 27.43918 |
| NOD 16-19 wks 2 A | mmu-miR-409-3p-4395443 | 23.27476 |
| NOD 16-19 wks 2 A | mmu-miR-434-3p-4395734 | 27.26279 |
| NOD 16-19 wks 2 A | mmu-miR-451-4373360 | 26.9762 |
| NOD 16-19 wks 2 A | mmu-miR-484-4381032 | 18.57567 |
| NOD 16-19 wks 2 A | mmu-miR-532-5p-4380928 | 28.37649 |
| NOD 16-19 wks 2 A | mmu-miR-574-3p-4395460 | 21.74607 |
| NOD 16-19 wks 2 A | mmu-miR-672-4395438 | 26.60971 |
| NOD 16-19 wks 2 A | mmu-miR-676-4386776 | 25.91983 |
| NOD 16-19 wks 2 A | mmu-miR-685-4386748 | 23.34101 |
| NOD 16-19 wks 2 A | mmu-miR-744-4395435 | 26.80767 |
| NOD 16-19 wks 2 A | mmu-miR-92a-4373013 | 21.24439 |
| NOD 16-19 wks 2 A | mmu-miR-93-4373302 | 26.9764 |
| NOD D 1 A | MammU6-4395470 | 25.74843 |
| NOD D 1 A | MammU6-4395470 | 25.94269 |
| NOD D 1 A | MammU6-4395470 | 26.00819 |
| NOD D 1 A | MammU6-4395470 | 26.01021 |
| NOD D 1 A | mmu-let-7c-4373167 | 35.4779 |
| NOD D 1 A | mmu-let-7g-4395393 | 30.8995 |
| NOD D 1 A | mmu-miR-106a-4395589 | 28.27223 |
| NOD D 1 A | mmu-miR-125a-5p-4395309 | 32.69894 |
| NOD D 1 A | mmu-miR-125b-5p-4373148 | 35.09808 |
| NOD D 1 A | mmu-miR-126-3p-4395339 | 28.93533 |
| NOD D 1 A | mmu-miR-126-5p-4373269 | 32.36127 |
| **Sample** | **Detector** | ***C*_T_** |
| NOD D 1 A | mmu-miR-133a-4395357 | 25.13507 |
| NOD D 1 A | mmu-miR-133b-4395358 | 30.62859 |
| NOD D 1 A | mmu-miR-134-4373299 | 34.39538 |
| NOD D 1 A | mmu-miR-139-5p-4395400 | 31.44473 |
| NOD D 1 A | mmu-miR-140-4373374 | 30.94412 |
| NOD D 1 A | mmu-miR-1-4395333 | 33.10138 |
| NOD D 1 A | mmu-miR-145-4395389 | 29.88918 |
| NOD D 1 A | mmu-miR-146a-4373132 | 28.5981 |
| NOD D 1 A | mmu-miR-146b-4373178 | 30.64235 |
| NOD D 1 A | mmu-miR-150-4373127 | 27.61091 |
| NOD D 1 A | mmu-miR-155-4395701 | 35.1206 |
| NOD D 1 A | mmu-miR-16-4373121 | 24.45257 |
| NOD D 1 A | mmu-miR-17-4395419 | 27.60848 |
| NOD D 1 A | mmu-miR-184-4373113 | 29.24862 |
| NOD D 1 A | mmu-miR-186-4395396 | 29.72774 |
| NOD D 1 A | mmu-miR-191-4395410 | 24.62788 |
| NOD D 1 A | mmu-miR-192-4373108 | 30.88659 |
| NOD D 1 A | mmu-miR-193b-4395597 | 29.80043 |
| NOD D 1 A | mmu-miR-195-4373105 | 31.36926 |
| NOD D 1 A | mmu-miR-199a-3p-4395415 | 33.54312 |
| NOD D 1 A | mmu-miR-19a-4373099 | 31.97012 |
| NOD D 1 A | mmu-miR-19b-4373098 | 28.67292 |
| NOD D 1 A | mmu-miR-200b-4395362 | 31.55392 |
| NOD D 1 A | mmu-miR-200c-4395411 | 33.20859 |
| NOD D 1 A | mmu-miR-203-4373095 | 32.63306 |
| NOD D 1 A | mmu-miR-204-4373094 | 32.68649 |
| NOD D 1 A | mmu-miR-20a-4373286 | 30.76227 |
| NOD D 1 A | mmu-miR-210-4373089 | 31.61872 |
| NOD D 1 A | mmu-miR-21-4373090 | 33.6189 |
| NOD D 1 A | mmu-miR-214-4395417 | 31.18757 |
| NOD D 1 A | mmu-miR-215-4373316 | 32.06926 |
| NOD D 1 A | mmu-miR-222-4395387 | 26.35537 |
| NOD D 1 A | mmu-miR-223-4395406 | 26.69797 |
| NOD D 1 A | mmu-miR-24-4373072 | 25.75048 |
| NOD D 1 A | mmu-miR-25-4373071 | 32.17836 |
| NOD D 1 A | mmu-miR-27a-4373287 | 34.68665 |
| NOD D 1 A | mmu-miR-296-5p-4373066 | 35.95978 |
| NOD D 1 A | mmu-miR-29a-4395223 | 30.14118 |
| NOD D 1 A | mmu-miR-29c-4395171 | 33.07595 |
| NOD D 1 A | mmu-miR-30a-4373061 | 31.79949 |
| NOD D 1 A | mmu-miR-30b-4373290 | 32.44556 |
| NOD D 1 A | mmu-miR-30c-4373060 | 32.27507 |
| NOD D 1 A | mmu-miR-30d-4373059 | 32.39983 |
| NOD D 1 A | mmu-miR-30e-4395334 | 31.31934 |
| NOD D 1 A | mmu-miR-320-4395388 | 28.22358 |
| NOD D 1 A | mmu-miR-324-3p-4395639 | 33.00365 |
| NOD D 1 A | mmu-miR-328-4373049 | 28.91172 |
| NOD D 1 A | mmu-miR-331-3p-4373046 | 33.44618 |
| NOD D 1 A | mmu-miR-339-3p-4395663 | 30.02033 |
| NOD D 1 A | mmu-miR-342-3p-4395371 | 30.56323 |
| NOD D 1 A | mmu-miR-365-4373194 | 34.95301 |
| NOD D 1 A | mmu-miR-375-4373027 | 34.52761 |
| NOD D 1 A | mmu-miR-409-3p-4395443 | 35.98288 |
| NOD D 1 A | mmu-miR-434-3p-4395734 | 36.34104 |
| NOD D 1 A | mmu-miR-451-4373360 | 32.31028 |
| NOD D 1 A | mmu-miR-484-4381032 | 24.82695 |
| NOD D 1 A | mmu-miR-532-5p-4380928 | 32.41592 |
| NOD D 1 A | mmu-miR-574-3p-4395460 | 28.2467 |
| NOD D 1 A | mmu-miR-672-4395438 | 33.25492 |
| NOD D 1 A | mmu-miR-676-4386776 | 32.83739 |
| NOD D 1 A | mmu-miR-685-4386748 | 29.43882 |
| **Sample** | **Detector** | ***C*_T_** |
| NOD D 1 A | mmu-miR-744-4395435 | 32.18225 |
| NOD D 1 A | mmu-miR-92a-4373013 | 29.26734 |
| NOD D 1 A | mmu-miR-93-4373302 | 30.59793 |
| NOD D 2 A | MammU6-4395470 | 20.8689 |
| NOD D 2 A | MammU6-4395470 | 21.1096 |
| NOD D 2 A | MammU6-4395470 | 21.23717 |
| NOD D 2 A | MammU6-4395470 | 21.26985 |
| NOD D 2 A | mmu-let-7c-4373167 | 28.78897 |
| NOD D 2 A | mmu-let-7g-4395393 | 28.46335 |
| NOD D 2 A | mmu-miR-106a-4395589 | 23.94266 |
| NOD D 2 A | mmu-miR-125a-5p-4395309 | 26.82837 |
| NOD D 2 A | mmu-miR-125b-5p-4373148 | 29.40075 |
| NOD D 2 A | mmu-miR-126-3p-4395339 | 23.52077 |
| NOD D 2 A | mmu-miR-126-5p-4373269 | 25.50025 |
| NOD D 2 A | mmu-miR-133a-4395357 | 19.34695 |
| NOD D 2 A | mmu-miR-133b-4395358 | 26.34054 |
| NOD D 2 A | mmu-miR-134-4373299 | 28.2828 |
| NOD D 2 A | mmu-miR-139-5p-4395400 | 23.91822 |
| NOD D 2 A | mmu-miR-140-4373374 | 27.19555 |
| NOD D 2 A | mmu-miR-1-4395333 | 29.15578 |
| NOD D 2 A | mmu-miR-145-4395389 | 23.67834 |
| NOD D 2 A | mmu-miR-146a-4373132 | 22.91104 |
| NOD D 2 A | mmu-miR-146b-4373178 | 25.87985 |
| NOD D 2 A | mmu-miR-150-4373127 | 20.09634 |
| NOD D 2 A | mmu-miR-155-4395701 | 28.93043 |
| NOD D 2 A | mmu-miR-16-4373121 | 20.27034 |
| NOD D 2 A | mmu-miR-17-4395419 | 24.27375 |
| NOD D 2 A | mmu-miR-184-4373113 | 27.22869 |
| NOD D 2 A | mmu-miR-186-4395396 | 24.81524 |
| NOD D 2 A | mmu-miR-191-4395410 | 18.96041 |
| NOD D 2 A | mmu-miR-192-4373108 | 23.62392 |
| NOD D 2 A | mmu-miR-193b-4395597 | 23.59004 |
| NOD D 2 A | mmu-miR-195-4373105 | 26.44166 |
| NOD D 2 A | mmu-miR-199a-3p-4395415 | 30.70584 |
| NOD D 2 A | mmu-miR-19a-4373099 | 27.9345 |
| NOD D 2 A | mmu-miR-19b-4373098 | 24.42781 |
| NOD D 2 A | mmu-miR-200b-4395362 | 28.72602 |
| NOD D 2 A | mmu-miR-200c-4395411 | 29.98302 |
| NOD D 2 A | mmu-miR-203-4373095 | 26.72946 |
| NOD D 2 A | mmu-miR-204-4373094 | 27.68337 |
| NOD D 2 A | mmu-miR-20a-4373286 | 25.98018 |
| NOD D 2 A | mmu-miR-210-4373089 | 27.00369 |
| NOD D 2 A | mmu-miR-21-4373090 | 28.01228 |
| NOD D 2 A | mmu-miR-214-4395417 | 27.58267 |
| NOD D 2 A | mmu-miR-215-4373316 | 26.83878 |
| NOD D 2 A | mmu-miR-222-4395387 | 21.72206 |
| NOD D 2 A | mmu-miR-223-4395406 | 20.88136 |
| NOD D 2 A | mmu-miR-24-4373072 | 20.97927 |
| NOD D 2 A | mmu-miR-25-4373071 | 28.68451 |
| NOD D 2 A | mmu-miR-27a-4373287 | 30.75121 |
| NOD D 2 A | mmu-miR-296-5p-4373066 | 31.00654 |
| NOD D 2 A | mmu-miR-29a-4395223 | 25.76381 |
| NOD D 2 A | mmu-miR-29c-4395171 | 26.22297 |
| NOD D 2 A | mmu-miR-30a-4373061 | 26.6674 |
| NOD D 2 A | mmu-miR-30b-4373290 | 26.73404 |
| NOD D 2 A | mmu-miR-30c-4373060 | 26.77606 |
| NOD D 2 A | mmu-miR-30d-4373059 | 28.09086 |
| NOD D 2 A | mmu-miR-30e-4395334 | 25.59891 |
| NOD D 2 A | mmu-miR-320-4395388 | 23.59032 |
| NOD D 2 A | mmu-miR-324-3p-4395639 | 26.11564 |
| NOD D 2 A | mmu-miR-328-4373049 | 22.75761 |
| **Sample** | **Detector** | ***C*_T_** |
| NOD D 2 A | mmu-miR-331-3p-4373046 | 28.15321 |
| NOD D 2 A | mmu-miR-339-3p-4395663 | 24.77438 |
| NOD D 2 A | mmu-miR-342-3p-4395371 | 23.83248 |
| NOD D 2 A | mmu-miR-365-4373194 | 28.58911 |
| NOD D 2 A | mmu-miR-375-4373027 | 25.7908 |
| NOD D 2 A | mmu-miR-409-3p-4395443 | 24.01968 |
| NOD D 2 A | mmu-miR-434-3p-4395734 | 27.94494 |
| NOD D 2 A | mmu-miR-451-4373360 | 27.81641 |
| NOD D 2 A | mmu-miR-484-4381032 | 18.94554 |
| NOD D 2 A | mmu-miR-532-5p-4380928 | 29.0169 |
| NOD D 2 A | mmu-miR-574-3p-4395460 | 21.66784 |
| NOD D 2 A | mmu-miR-672-4395438 | 26.71899 |
| NOD D 2 A | mmu-miR-676-4386776 | 26.97718 |
| NOD D 2 A | mmu-miR-685-4386748 | 24.78186 |
| NOD D 2 A | mmu-miR-744-4395435 | 27.46572 |
| NOD D 2 A | mmu-miR-92a-4373013 | 20.92882 |
| NOD D 2 A | mmu-miR-93-4373302 | 27.98998 |
| NOR 3-4 wks 1 A | MammU6-4395470 | 16.92925 |
| NOR 3-4 wks 1 A | MammU6-4395470 | 16.96744 |
| NOR 3-4 wks 1 A | MammU6-4395470 | 17.00372 |
| NOR 3-4 wks 1 A | MammU6-4395470 | 17.23107 |
| NOR 3-4 wks 1 A | mmu-let-7c-4373167 | 19.56808 |
| NOR 3-4 wks 1 A | mmu-let-7g-4395393 | 21.00023 |
| NOR 3-4 wks 1 A | mmu-miR-106a-4395589 | 14.83661 |
| NOR 3-4 wks 1 A | mmu-miR-125a-5p-4395309 | 21.49051 |
| NOR 3-4 wks 1 A | mmu-miR-125b-5p-4373148 | 20.2046 |
| NOR 3-4 wks 1 A | mmu-miR-126-3p-4395339 | 17.4183 |
| NOR 3-4 wks 1 A | mmu-miR-126-5p-4373269 | 18.59359 |
| NOR 3-4 wks 1 A | mmu-miR-133a-4395357 | 16.6178 |
| NOR 3-4 wks 1 A | mmu-miR-133b-4395358 | 19.61181 |
| NOR 3-4 wks 1 A | mmu-miR-134-4373299 | 22.70875 |
| NOR 3-4 wks 1 A | mmu-miR-139-5p-4395400 | 19.41284 |
| NOR 3-4 wks 1 A | mmu-miR-140-4373374 | 17.95464 |
| NOR 3-4 wks 1 A | mmu-miR-1-4395333 | 21.31604 |
| NOR 3-4 wks 1 A | mmu-miR-145-4395389 | 17.96854 |
| NOR 3-4 wks 1 A | mmu-miR-146a-4373132 | 16.76853 |
| NOR 3-4 wks 1 A | mmu-miR-146b-4373178 | 19.27383 |
| NOR 3-4 wks 1 A | mmu-miR-150-4373127 | 15.74049 |
| NOR 3-4 wks 1 A | mmu-miR-155-4395701 | 22.49262 |
| NOR 3-4 wks 1 A | mmu-miR-16-4373121 | 13.38053 |
| NOR 3-4 wks 1 A | mmu-miR-17-4395419 | 14.66477 |
| NOR 3-4 wks 1 A | mmu-miR-184-4373113 | 25.40616 |
| NOR 3-4 wks 1 A | mmu-miR-186-4395396 | 18.59364 |
| NOR 3-4 wks 1 A | mmu-miR-191-4395410 | 13.064 |
| NOR 3-4 wks 1 A | mmu-miR-192-4373108 | 18.2016 |
| NOR 3-4 wks 1 A | mmu-miR-193b-4395597 | 18.85567 |
| NOR 3-4 wks 1 A | mmu-miR-195-4373105 | 18.97531 |
| NOR 3-4 wks 1 A | mmu-miR-199a-3p-4395415 | 21.14139 |
| NOR 3-4 wks 1 A | mmu-miR-19a-4373099 | 17.94188 |
| NOR 3-4 wks 1 A | mmu-miR-19b-4373098 | 14.31476 |
| NOR 3-4 wks 1 A | mmu-miR-200b-4395362 | 20.73595 |
| NOR 3-4 wks 1 A | mmu-miR-200c-4395411 | 22.19069 |
| NOR 3-4 wks 1 A | mmu-miR-203-4373095 | 20.30065 |
| NOR 3-4 wks 1 A | mmu-miR-204-4373094 | 24.22087 |
| NOR 3-4 wks 1 A | mmu-miR-20a-4373286 | 14.97558 |
| NOR 3-4 wks 1 A | mmu-miR-210-4373089 | 19.79775 |
| NOR 3-4 wks 1 A | mmu-miR-21-4373090 | 17.50778 |
| NOR 3-4 wks 1 A | mmu-miR-214-4395417 | 20.28608 |
| NOR 3-4 wks 1 A | mmu-miR-215-4373316 | 19.8592 |
| NOR 3-4 wks 1 A | mmu-miR-222-4395387 | 15.95731 |
| NOR 3-4 wks 1 A | mmu-miR-223-4395406 | 14.24063 |
| **Sample** | **Detector** | ***C*_T_** |
| NOR 3-4 wks 1 A | mmu-miR-24-4373072 | 14.57258 |
| NOR 3-4 wks 1 A | mmu-miR-25-4373071 | 18.59546 |
| NOR 3-4 wks 1 A | mmu-miR-27a-4373287 | 22.83946 |
| NOR 3-4 wks 1 A | mmu-miR-296-5p-4373066 | 20.51428 |
| NOR 3-4 wks 1 A | mmu-miR-29a-4395223 | 19.31304 |
| NOR 3-4 wks 1 A | mmu-miR-29c-4395171 | 23.35774 |
| NOR 3-4 wks 1 A | mmu-miR-30a-4373061 | 18.58693 |
| NOR 3-4 wks 1 A | mmu-miR-30b-4373290 | 17.13706 |
| NOR 3-4 wks 1 A | mmu-miR-30c-4373060 | 16.98157 |
| NOR 3-4 wks 1 A | mmu-miR-30d-4373059 | 19.94271 |
| NOR 3-4 wks 1 A | mmu-miR-30e-4395334 | 19.92218 |
| NOR 3-4 wks 1 A | mmu-miR-320-4395388 | 19.12652 |
| NOR 3-4 wks 1 A | mmu-miR-324-3p-4395639 | 21.47784 |
| NOR 3-4 wks 1 A | mmu-miR-328-4373049 | 17.43113 |
| NOR 3-4 wks 1 A | mmu-miR-331-3p-4373046 | 22.95987 |
| NOR 3-4 wks 1 A | mmu-miR-339-3p-4395663 | 21.33787 |
| NOR 3-4 wks 1 A | mmu-miR-342-3p-4395371 | 19.41079 |
| NOR 3-4 wks 1 A | mmu-miR-365-4373194 | 22.35415 |
| NOR 3-4 wks 1 A | mmu-miR-375-4373027 | 20.10263 |
| NOR 3-4 wks 1 A | mmu-miR-409-3p-4395443 | 22.69764 |
| NOR 3-4 wks 1 A | mmu-miR-434-3p-4395734 | 22.58578 |
| NOR 3-4 wks 1 A | mmu-miR-451-4373360 | 15.74082 |
| NOR 3-4 wks 1 A | mmu-miR-484-4381032 | 14.99389 |
| NOR 3-4 wks 1 A | mmu-miR-532-5p-4380928 | 21.34415 |
| NOR 3-4 wks 1 A | mmu-miR-574-3p-4395460 | 16.11998 |
| NOR 3-4 wks 1 A | mmu-miR-672-4395438 | 21.23011 |
| NOR 3-4 wks 1 A | mmu-miR-676-4386776 | 22.54601 |
| NOR 3-4 wks 1 A | mmu-miR-685-4386748 | 19.2227 |
| NOR 3-4 wks 1 A | mmu-miR-744-4395435 | 20.98208 |
| NOR 3-4 wks 1 A | mmu-miR-92a-4373013 | 15.66244 |
| NOR 3-4 wks 1 A | mmu-miR-93-4373302 | 16.95392 |
| NOR 3-4 wks 2 A | MammU6-4395470 | 16.70015 |
| NOR 3-4 wks 2 A | MammU6-4395470 | 16.77499 |
| NOR 3-4 wks 2 A | MammU6-4395470 | 16.82459 |
| NOR 3-4 wks 2 A | MammU6-4395470 | 16.86423 |
| NOR 3-4 wks 2 A | mmu-let-7c-4373167 | 19.3359 |
| NOR 3-4 wks 2 A | mmu-let-7g-4395393 | 20.9511 |
| NOR 3-4 wks 2 A | mmu-miR-106a-4395589 | 14.75387 |
| NOR 3-4 wks 2 A | mmu-miR-125a-5p-4395309 | 21.18636 |
| NOR 3-4 wks 2 A | mmu-miR-125b-5p-4373148 | 18.95271 |
| NOR 3-4 wks 2 A | mmu-miR-126-3p-4395339 | 18.06271 |
| NOR 3-4 wks 2 A | mmu-miR-126-5p-4373269 | 18.95845 |
| NOR 3-4 wks 2 A | mmu-miR-133a-4395357 | 15.96423 |
| NOR 3-4 wks 2 A | mmu-miR-133b-4395358 | 19.21437 |
| NOR 3-4 wks 2 A | mmu-miR-134-4373299 | 23.09719 |
| NOR 3-4 wks 2 A | mmu-miR-139-5p-4395400 | 19.46659 |
| NOR 3-4 wks 2 A | mmu-miR-140-4373374 | 18.62604 |
| NOR 3-4 wks 2 A | mmu-miR-1-4395333 | 20.95793 |
| NOR 3-4 wks 2 A | mmu-miR-145-4395389 | 17.49112 |
| NOR 3-4 wks 2 A | mmu-miR-146a-4373132 | 17.56478 |
| NOR 3-4 wks 2 A | mmu-miR-146b-4373178 | 20.26701 |
| NOR 3-4 wks 2 A | mmu-miR-150-4373127 | 15.83312 |
| NOR 3-4 wks 2 A | mmu-miR-155-4395701 | 23.77452 |
| NOR 3-4 wks 2 A | mmu-miR-16-4373121 | 13.61804 |
| NOR 3-4 wks 2 A | mmu-miR-17-4395419 | 14.81457 |
| NOR 3-4 wks 2 A | mmu-miR-184-4373113 | 25.46198 |
| NOR 3-4 wks 2 A | mmu-miR-186-4395396 | 19.66573 |
| NOR 3-4 wks 2 A | mmu-miR-191-4395410 | 12.97889 |
| NOR 3-4 wks 2 A | mmu-miR-192-4373108 | 17.94218 |
| NOR 3-4 wks 2 A | mmu-miR-193b-4395597 | 18.55205 |
| NOR 3-4 wks 2 A | mmu-miR-195-4373105 | 18.98403 |
| **Sample** | **Detector** | ***C*_T_** |
| NOR 3-4 wks 2 A | mmu-miR-199a-3p-4395415 | 21.59147 |
| NOR 3-4 wks 2 A | mmu-miR-19a-4373099 | 18.51062 |
| NOR 3-4 wks 2 A | mmu-miR-19b-4373098 | 14.70773 |
| NOR 3-4 wks 2 A | mmu-miR-200b-4395362 | 21.99975 |
| NOR 3-4 wks 2 A | mmu-miR-200c-4395411 | 23.00569 |
| NOR 3-4 wks 2 A | mmu-miR-203-4373095 | 19.94539 |
| NOR 3-4 wks 2 A | mmu-miR-204-4373094 | 23.62648 |
| NOR 3-4 wks 2 A | mmu-miR-20a-4373286 | 14.73916 |
| NOR 3-4 wks 2 A | mmu-miR-210-4373089 | 19.99854 |
| NOR 3-4 wks 2 A | mmu-miR-21-4373090 | 16.98281 |
| NOR 3-4 wks 2 A | mmu-miR-214-4395417 | 19.96821 |
| NOR 3-4 wks 2 A | mmu-miR-215-4373316 | 19.96289 |
| NOR 3-4 wks 2 A | mmu-miR-222-4395387 | 16.42954 |
| NOR 3-4 wks 2 A | mmu-miR-223-4395406 | 13.95265 |
| NOR 3-4 wks 2 A | mmu-miR-24-4373072 | 14.73144 |
| NOR 3-4 wks 2 A | mmu-miR-25-4373071 | 17.30637 |
| NOR 3-4 wks 2 A | mmu-miR-27a-4373287 | 22.63499 |
| NOR 3-4 wks 2 A | mmu-miR-296-5p-4373066 | 19.16554 |
| NOR 3-4 wks 2 A | mmu-miR-29a-4395223 | 19.23224 |
| NOR 3-4 wks 2 A | mmu-miR-29c-4395171 | 22.9596 |
| NOR 3-4 wks 2 A | mmu-miR-30a-4373061 | 17.80967 |
| NOR 3-4 wks 2 A | mmu-miR-30b-4373290 | 16.09057 |
| NOR 3-4 wks 2 A | mmu-miR-30c-4373060 | 16.3482 |
| NOR 3-4 wks 2 A | mmu-miR-30d-4373059 | 19.15254 |
| NOR 3-4 wks 2 A | mmu-miR-30e-4395334 | 19.84654 |
| NOR 3-4 wks 2 A | mmu-miR-320-4395388 | 18.97035 |
| NOR 3-4 wks 2 A | mmu-miR-324-3p-4395639 | 20.42232 |
| NOR 3-4 wks 2 A | mmu-miR-328-4373049 | 15.8719 |
| NOR 3-4 wks 2 A | mmu-miR-331-3p-4373046 | 22.97559 |
| NOR 3-4 wks 2 A | mmu-miR-339-3p-4395663 | 21.97455 |
| NOR 3-4 wks 2 A | mmu-miR-342-3p-4395371 | 19.69796 |
| NOR 3-4 wks 2 A | mmu-miR-365-4373194 | 21.0516 |
| NOR 3-4 wks 2 A | mmu-miR-375-4373027 | 20.24352 |
| NOR 3-4 wks 2 A | mmu-miR-409-3p-4395443 | 22.48363 |
| NOR 3-4 wks 2 A | mmu-miR-434-3p-4395734 | 21.72971 |
| NOR 3-4 wks 2 A | mmu-miR-451-4373360 | 14.95347 |
| NOR 3-4 wks 2 A | mmu-miR-484-4381032 | 14.97871 |
| NOR 3-4 wks 2 A | mmu-miR-532-5p-4380928 | 21.25091 |
| NOR 3-4 wks 2 A | mmu-miR-574-3p-4395460 | 16.3444 |
| NOR 3-4 wks 2 A | mmu-miR-672-4395438 | 21.80893 |
| NOR 3-4 wks 2 A | mmu-miR-676-4386776 | 22.54912 |
| NOR 3-4 wks 2 A | mmu-miR-685-4386748 | 19.00197 |
| NOR 3-4 wks 2 A | mmu-miR-744-4395435 | 20.14569 |
| NOR 3-4 wks 2 A | mmu-miR-92a-4373013 | 14.4875 |
| NOR 3-4 wks 2 A | mmu-miR-93-4373302 | 16.72907 |
| NOR 7-8 wks 1 A | MammU6-4395470 | 18.64387 |
| NOR 7-8 wks 1 A | MammU6-4395470 | 18.67999 |
| NOR 7-8 wks 1 A | MammU6-4395470 | 18.75944 |
| NOR 7-8 wks 1 A | MammU6-4395470 | 18.86109 |
| NOR 7-8 wks 1 A | mmu-let-7c-4373167 | 18.94599 |
| NOR 7-8 wks 1 A | mmu-let-7g-4395393 | 21.59773 |
| NOR 7-8 wks 1 A | mmu-miR-106a-4395589 | 15.49769 |
| NOR 7-8 wks 1 A | mmu-miR-125a-5p-4395309 | 20.43344 |
| NOR 7-8 wks 1 A | mmu-miR-125b-5p-4373148 | 19.93908 |
| NOR 7-8 wks 1 A | mmu-miR-126-3p-4395339 | 15.64905 |
| NOR 7-8 wks 1 A | mmu-miR-126-5p-4373269 | 17.82369 |
| NOR 7-8 wks 1 A | mmu-miR-133a-4395357 | 15.62042 |
| NOR 7-8 wks 1 A | mmu-miR-133b-4395358 | 19.17351 |
| NOR 7-8 wks 1 A | mmu-miR-134-4373299 | 20.98318 |
| NOR 7-8 wks 1 A | mmu-miR-139-5p-4395400 | 18.75719 |
| NOR 7-8 wks 1 A | mmu-miR-140-4373374 | 18.02569 |
| **Sample** | **Detector** | ***C*_T_** |
| NOR 7-8 wks 1 A | mmu-miR-1-4395333 | 20.45256 |
| NOR 7-8 wks 1 A | mmu-miR-145-4395389 | 17.38423 |
| NOR 7-8 wks 1 A | mmu-miR-146a-4373132 | 16.03176 |
| NOR 7-8 wks 1 A | mmu-miR-146b-4373178 | 18.2843 |
| NOR 7-8 wks 1 A | mmu-miR-150-4373127 | 15.2982 |
| NOR 7-8 wks 1 A | mmu-miR-155-4395701 | 21.37164 |
| NOR 7-8 wks 1 A | mmu-miR-16-4373121 | 13.92366 |
| NOR 7-8 wks 1 A | mmu-miR-17-4395419 | 15.5506 |
| NOR 7-8 wks 1 A | mmu-miR-184-4373113 | 25.08546 |
| NOR 7-8 wks 1 A | mmu-miR-186-4395396 | 18.51575 |
| NOR 7-8 wks 1 A | mmu-miR-191-4395410 | 13.29182 |
| NOR 7-8 wks 1 A | mmu-miR-192-4373108 | 17.81092 |
| NOR 7-8 wks 1 A | mmu-miR-193b-4395597 | 19.03829 |
| NOR 7-8 wks 1 A | mmu-miR-195-4373105 | 19.4353 |
| NOR 7-8 wks 1 A | mmu-miR-199a-3p-4395415 | 20.92381 |
| NOR 7-8 wks 1 A | mmu-miR-19a-4373099 | 18.24368 |
| NOR 7-8 wks 1 A | mmu-miR-19b-4373098 | 14.57133 |
| NOR 7-8 wks 1 A | mmu-miR-200b-4395362 | 19.4792 |
| NOR 7-8 wks 1 A | mmu-miR-200c-4395411 | 20.23038 |
| NOR 7-8 wks 1 A | mmu-miR-203-4373095 | 21.25856 |
| NOR 7-8 wks 1 A | mmu-miR-204-4373094 | 23.56139 |
| NOR 7-8 wks 1 A | mmu-miR-20a-4373286 | 16.08418 |
| NOR 7-8 wks 1 A | mmu-miR-210-4373089 | 20.45682 |
| NOR 7-8 wks 1 A | mmu-miR-21-4373090 | 18.17655 |
| NOR 7-8 wks 1 A | mmu-miR-214-4395417 | 20.94282 |
| NOR 7-8 wks 1 A | mmu-miR-215-4373316 | 19.69053 |
| NOR 7-8 wks 1 A | mmu-miR-222-4395387 | 14.92205 |
| NOR 7-8 wks 1 A | mmu-miR-223-4395406 | 14.02694 |
| NOR 7-8 wks 1 A | mmu-miR-24-4373072 | 13.99294 |
| NOR 7-8 wks 1 A | mmu-miR-25-4373071 | 19.93145 |
| NOR 7-8 wks 1 A | mmu-miR-27a-4373287 | 22.70029 |
| NOR 7-8 wks 1 A | mmu-miR-296-5p-4373066 | 22.57318 |
| NOR 7-8 wks 1 A | mmu-miR-29a-4395223 | 18.36103 |
| NOR 7-8 wks 1 A | mmu-miR-29c-4395171 | 22.3256 |
| NOR 7-8 wks 1 A | mmu-miR-30a-4373061 | 18.27992 |
| NOR 7-8 wks 1 A | mmu-miR-30b-4373290 | 17.11497 |
| NOR 7-8 wks 1 A | mmu-miR-30c-4373060 | 16.93386 |
| NOR 7-8 wks 1 A | mmu-miR-30d-4373059 | 20.01515 |
| NOR 7-8 wks 1 A | mmu-miR-30e-4395334 | 18.76382 |
| NOR 7-8 wks 1 A | mmu-miR-320-4395388 | 19.48062 |
| NOR 7-8 wks 1 A | mmu-miR-324-3p-4395639 | 22.07725 |
| NOR 7-8 wks 1 A | mmu-miR-328-4373049 | 16.76901 |
| NOR 7-8 wks 1 A | mmu-miR-331-3p-4373046 | 22.11653 |
| NOR 7-8 wks 1 A | mmu-miR-339-3p-4395663 | 19.92013 |
| NOR 7-8 wks 1 A | mmu-miR-342-3p-4395371 | 18.96146 |
| NOR 7-8 wks 1 A | mmu-miR-365-4373194 | 21.57359 |
| NOR 7-8 wks 1 A | mmu-miR-375-4373027 | 19.19935 |
| NOR 7-8 wks 1 A | mmu-miR-409-3p-4395443 | 22.63292 |
| NOR 7-8 wks 1 A | mmu-miR-434-3p-4395734 | 22.86275 |
| NOR 7-8 wks 1 A | mmu-miR-451-4373360 | 17.24482 |
| NOR 7-8 wks 1 A | mmu-miR-484-4381032 | 14.53303 |
| NOR 7-8 wks 1 A | mmu-miR-532-5p-4380928 | 21.29947 |
| NOR 7-8 wks 1 A | mmu-miR-574-3p-4395460 | 16.30972 |
| NOR 7-8 wks 1 A | mmu-miR-672-4395438 | 20.98898 |
| NOR 7-8 wks 1 A | mmu-miR-676-4386776 | 21.75868 |
| NOR 7-8 wks 1 A | mmu-miR-685-4386748 | 21.36303 |
| NOR 7-8 wks 1 A | mmu-miR-744-4395435 | 21.79086 |
| NOR 7-8 wks 1 A | mmu-miR-92a-4373013 | 16.00717 |
| NOR 7-8 wks 1 A | mmu-miR-93-4373302 | 18.02354 |
| NOR 7-8 wks 2 A | MammU6-4395470 | 17.48757 |
| NOR 7-8 wks 2 A | MammU6-4395470 | 17.59064 |
| **Sample** | **Detector** | ***C*_T_** |
| NOR 7-8 wks 2 A | MammU6-4395470 | 17.7311 |
| NOR 7-8 wks 2 A | MammU6-4395470 | 17.78536 |
| NOR 7-8 wks 2 A | mmu-let-7c-4373167 | 18.87183 |
| NOR 7-8 wks 2 A | mmu-let-7g-4395393 | 21.49566 |
| NOR 7-8 wks 2 A | mmu-miR-106a-4395589 | 15.5826 |
| NOR 7-8 wks 2 A | mmu-miR-125a-5p-4395309 | 20.51344 |
| NOR 7-8 wks 2 A | mmu-miR-125b-5p-4373148 | 18.3945 |
| NOR 7-8 wks 2 A | mmu-miR-126-3p-4395339 | 16.57743 |
| NOR 7-8 wks 2 A | mmu-miR-126-5p-4373269 | 17.82886 |
| NOR 7-8 wks 2 A | mmu-miR-133a-4395357 | 14.87433 |
| NOR 7-8 wks 2 A | mmu-miR-133b-4395358 | 18.24586 |
| NOR 7-8 wks 2 A | mmu-miR-134-4373299 | 21.37911 |
| NOR 7-8 wks 2 A | mmu-miR-139-5p-4395400 | 18.92746 |
| NOR 7-8 wks 2 A | mmu-miR-140-4373374 | 19.0639 |
| NOR 7-8 wks 2 A | mmu-miR-1-4395333 | 19.37142 |
| NOR 7-8 wks 2 A | mmu-miR-145-4395389 | 16.54775 |
| NOR 7-8 wks 2 A | mmu-miR-146a-4373132 | 16.95765 |
| NOR 7-8 wks 2 A | mmu-miR-146b-4373178 | 19.80222 |
| NOR 7-8 wks 2 A | mmu-miR-150-4373127 | 14.58566 |
| NOR 7-8 wks 2 A | mmu-miR-155-4395701 | 22.91728 |
| NOR 7-8 wks 2 A | mmu-miR-16-4373121 | 14.13461 |
| NOR 7-8 wks 2 A | mmu-miR-17-4395419 | 15.7793 |
| NOR 7-8 wks 2 A | mmu-miR-184-4373113 | 24.38505 |
| NOR 7-8 wks 2 A | mmu-miR-186-4395396 | 20.70569 |
| NOR 7-8 wks 2 A | mmu-miR-191-4395410 | 12.94791 |
| NOR 7-8 wks 2 A | mmu-miR-192-4373108 | 17.04655 |
| NOR 7-8 wks 2 A | mmu-miR-193b-4395597 | 18.47094 |
| NOR 7-8 wks 2 A | mmu-miR-195-4373105 | 19.47177 |
| NOR 7-8 wks 2 A | mmu-miR-199a-3p-4395415 | 21.61736 |
| NOR 7-8 wks 2 A | mmu-miR-19a-4373099 | 18.50294 |
| NOR 7-8 wks 2 A | mmu-miR-19b-4373098 | 14.11036 |
| NOR 7-8 wks 2 A | mmu-miR-200b-4395362 | 20.0324 |
| NOR 7-8 wks 2 A | mmu-miR-200c-4395411 | 21.95532 |
| NOR 7-8 wks 2 A | mmu-miR-203-4373095 | 20.33614 |
| NOR 7-8 wks 2 A | mmu-miR-204-4373094 | 22.71426 |
| NOR 7-8 wks 2 A | mmu-miR-20a-4373286 | 15.49203 |
| NOR 7-8 wks 2 A | mmu-miR-210-4373089 | 19.94304 |
| NOR 7-8 wks 2 A | mmu-miR-21-4373090 | 17.54451 |
| NOR 7-8 wks 2 A | mmu-miR-214-4395417 | 20.49023 |
| NOR 7-8 wks 2 A | mmu-miR-215-4373316 | 19.31869 |
| NOR 7-8 wks 2 A | mmu-miR-222-4395387 | 15.93476 |
| NOR 7-8 wks 2 A | mmu-miR-223-4395406 | 13.66824 |
| NOR 7-8 wks 2 A | mmu-miR-24-4373072 | 13.98134 |
| NOR 7-8 wks 2 A | mmu-miR-25-4373071 | 18.10728 |
| NOR 7-8 wks 2 A | mmu-miR-27a-4373287 | 21.83226 |
| NOR 7-8 wks 2 A | mmu-miR-296-5p-4373066 | 20.26786 |
| NOR 7-8 wks 2 A | mmu-miR-29a-4395223 | 18.21936 |
| NOR 7-8 wks 2 A | mmu-miR-29c-4395171 | 22.0376 |
| NOR 7-8 wks 2 A | mmu-miR-30a-4373061 | 16.92994 |
| NOR 7-8 wks 2 A | mmu-miR-30b-4373290 | 15.91224 |
| NOR 7-8 wks 2 A | mmu-miR-30c-4373060 | 16.46066 |
| NOR 7-8 wks 2 A | mmu-miR-30d-4373059 | 18.43761 |
| NOR 7-8 wks 2 A | mmu-miR-30e-4395334 | 18.63112 |
| NOR 7-8 wks 2 A | mmu-miR-320-4395388 | 19.28489 |
| NOR 7-8 wks 2 A | mmu-miR-324-3p-4395639 | 21.3716 |
| NOR 7-8 wks 2 A | mmu-miR-328-4373049 | 14.68555 |
| NOR 7-8 wks 2 A | mmu-miR-331-3p-4373046 | 22.03442 |
| NOR 7-8 wks 2 A | mmu-miR-339-3p-4395663 | 21.0672 |
| NOR 7-8 wks 2 A | mmu-miR-342-3p-4395371 | 19.03114 |
| NOR 7-8 wks 2 A | mmu-miR-365-4373194 | 20.6254 |
| NOR 7-8 wks 2 A | mmu-miR-375-4373027 | 17.9605 |
| **Sample** | **Detector** | ***C*_T_** |
| NOR 7-8 wks 2 A | mmu-miR-409-3p-4395443 | 21.76475 |
| NOR 7-8 wks 2 A | mmu-miR-434-3p-4395734 | 21.93215 |
| NOR 7-8 wks 2 A | mmu-miR-451-4373360 | 16.76444 |
| NOR 7-8 wks 2 A | mmu-miR-484-4381032 | 14.7208 |
| NOR 7-8 wks 2 A | mmu-miR-532-5p-4380928 | 20.78355 |
| NOR 7-8 wks 2 A | mmu-miR-574-3p-4395460 | 16.57406 |
| NOR 7-8 wks 2 A | mmu-miR-672-4395438 | 22.2641 |
| NOR 7-8 wks 2 A | mmu-miR-676-4386776 | 21.21393 |
| NOR 7-8 wks 2 A | mmu-miR-685-4386748 | 18.57062 |
| NOR 7-8 wks 2 A | mmu-miR-744-4395435 | 20.438 |
| NOR 7-8 wks 2 A | mmu-miR-92a-4373013 | 14.01367 |
| NOR 7-8 wks 2 A | mmu-miR-93-4373302 | 17.75479 |
| NOR 16-19 wks 1 A | MammU6-4395470 | 18.0935 |
| NOR 16-19 wks 1 A | MammU6-4395470 | 18.17799 |
| NOR 16-19 wks 1 A | MammU6-4395470 | 18.41444 |
| NOR 16-19 wks 1 A | MammU6-4395470 | 18.56132 |
| NOR 16-19 wks 1 A | mmu-let-7c-4373167 | 19.39743 |
| NOR 16-19 wks 1 A | mmu-let-7g-4395393 | 20.83402 |
| NOR 16-19 wks 1 A | mmu-miR-106a-4395589 | 15.20496 |
| NOR 16-19 wks 1 A | mmu-miR-125a-5p-4395309 | 22.48056 |
| NOR 16-19 wks 1 A | mmu-miR-125b-5p-4373148 | 19.20043 |
| NOR 16-19 wks 1 A | mmu-miR-126-3p-4395339 | 16.68203 |
| NOR 16-19 wks 1 A | mmu-miR-126-5p-4373269 | 18.44341 |
| NOR 16-19 wks 1 A | mmu-miR-133a-4395357 | 15.33758 |
| NOR 16-19 wks 1 A | mmu-miR-133b-4395358 | 18.34691 |
| NOR 16-19 wks 1 A | mmu-miR-134-4373299 | 21.79839 |
| NOR 16-19 wks 1 A | mmu-miR-139-5p-4395400 | 18.8109 |
| NOR 16-19 wks 1 A | mmu-miR-140-4373374 | 18.22067 |
| NOR 16-19 wks 1 A | mmu-miR-1-4395333 | 18.70452 |
| NOR 16-19 wks 1 A | mmu-miR-145-4395389 | 17.19831 |
| NOR 16-19 wks 1 A | mmu-miR-146a-4373132 | 16.44616 |
| NOR 16-19 wks 1 A | mmu-miR-146b-4373178 | 19.26832 |
| NOR 16-19 wks 1 A | mmu-miR-150-4373127 | 14.58005 |
| NOR 16-19 wks 1 A | mmu-miR-155-4395701 | 23.61344 |
| NOR 16-19 wks 1 A | mmu-miR-16-4373121 | 13.14141 |
| NOR 16-19 wks 1 A | mmu-miR-17-4395419 | 15.30913 |
| NOR 16-19 wks 1 A | mmu-miR-184-4373113 | 24.73421 |
| NOR 16-19 wks 1 A | mmu-miR-186-4395396 | 20.46784 |
| NOR 16-19 wks 1 A | mmu-miR-191-4395410 | 12.58745 |
| NOR 16-19 wks 1 A | mmu-miR-192-4373108 | 16.47775 |
| NOR 16-19 wks 1 A | mmu-miR-193b-4395597 | 18.34038 |
| NOR 16-19 wks 1 A | mmu-miR-195-4373105 | 19.41697 |
| NOR 16-19 wks 1 A | mmu-miR-199a-3p-4395415 | 21.89666 |
| NOR 16-19 wks 1 A | mmu-miR-19a-4373099 | 17.06596 |
| NOR 16-19 wks 1 A | mmu-miR-19b-4373098 | 13.25965 |
| NOR 16-19 wks 1 A | mmu-miR-200b-4395362 | 19.9538 |
| NOR 16-19 wks 1 A | mmu-miR-200c-4395411 | 22.54078 |
| NOR 16-19 wks 1 A | mmu-miR-203-4373095 | 20.23838 |
| NOR 16-19 wks 1 A | mmu-miR-204-4373094 | 24.598 |
| NOR 16-19 wks 1 A | mmu-miR-20a-4373286 | 15.15873 |
| NOR 16-19 wks 1 A | mmu-miR-210-4373089 | 19.53945 |
| NOR 16-19 wks 1 A | mmu-miR-21-4373090 | 16.20235 |
| NOR 16-19 wks 1 A | mmu-miR-214-4395417 | 21.42518 |
| NOR 16-19 wks 1 A | mmu-miR-215-4373316 | 19.08094 |
| NOR 16-19 wks 1 A | mmu-miR-222-4395387 | 15.65315 |
| NOR 16-19 wks 1 A | mmu-miR-223-4395406 | 12.87714 |
| NOR 16-19 wks 1 A | mmu-miR-24-4373072 | 13.96351 |
| NOR 16-19 wks 1 A | mmu-miR-25-4373071 | 18.22614 |
| NOR 16-19 wks 1 A | mmu-miR-27a-4373287 | 20.67082 |
| NOR 16-19 wks 1 A | mmu-miR-296-5p-4373066 | 22.53693 |
| NOR 16-19 wks 1 A | mmu-miR-29a-4395223 | 16.80028 |
| **Sample** | **Detector** | ***C*_T_** |
| NOR 16-19 wks 1 A | mmu-miR-29c-4395171 | 22.37636 |
| NOR 16-19 wks 1 A | mmu-miR-30a-4373061 | 17.21588 |
| NOR 16-19 wks 1 A | mmu-miR-30b-4373290 | 15.51527 |
| NOR 16-19 wks 1 A | mmu-miR-30c-4373060 | 16.41876 |
| NOR 16-19 wks 1 A | mmu-miR-30d-4373059 | 18.8584 |
| NOR 16-19 wks 1 A | mmu-miR-30e-4395334 | 18.40535 |
| NOR 16-19 wks 1 A | mmu-miR-320-4395388 | 19.04791 |
| NOR 16-19 wks 1 A | mmu-miR-324-3p-4395639 | 21.25114 |
| NOR 16-19 wks 1 A | mmu-miR-328-4373049 | 15.01314 |
| NOR 16-19 wks 1 A | mmu-miR-331-3p-4373046 | 22.94491 |
| NOR 16-19 wks 1 A | mmu-miR-339-3p-4395663 | 21.09046 |
| NOR 16-19 wks 1 A | mmu-miR-342-3p-4395371 | 18.95755 |
| NOR 16-19 wks 1 A | mmu-miR-365-4373194 | 20.35218 |
| NOR 16-19 wks 1 A | mmu-miR-375-4373027 | 18.00879 |
| NOR 16-19 wks 1 A | mmu-miR-409-3p-4395443 | 22.1024 |
| NOR 16-19 wks 1 A | mmu-miR-434-3p-4395734 | 22.13993 |
| NOR 16-19 wks 1 A | mmu-miR-451-4373360 | 16.17584 |
| NOR 16-19 wks 1 A | mmu-miR-484-4381032 | 14.88984 |
| NOR 16-19 wks 1 A | mmu-miR-532-5p-4380928 | 20.51955 |
| NOR 16-19 wks 1 A | mmu-miR-574-3p-4395460 | 17.23519 |
| NOR 16-19 wks 1 A | mmu-miR-672-4395438 | 23.45274 |
| NOR 16-19 wks 1 A | mmu-miR-676-4386776 | 21.42612 |
| NOR 16-19 wks 1 A | mmu-miR-685-4386748 | 18.76505 |
| NOR 16-19 wks 1 A | mmu-miR-744-4395435 | 20.38569 |
| NOR 16-19 wks 1 A | mmu-miR-92a-4373013 | 14.52167 |
| NOR 16-19 wks 1 A | mmu-miR-93-4373302 | 17.45858 |
| NOR 16-19 wks 2 A | MammU6-4395470 | 16.86893 |
| NOR 16-19 wks 2 A | MammU6-4395470 | 16.90907 |
| NOR 16-19 wks 2 A | MammU6-4395470 | 16.94351 |
| NOR 16-19 wks 2 A | MammU6-4395470 | 16.97378 |
| NOR 16-19 wks 2 A | mmu-let-7c-4373167 | 18.94564 |
| NOR 16-19 wks 2 A | mmu-let-7g-4395393 | 20.78197 |
| NOR 16-19 wks 2 A | mmu-miR-106a-4395589 | 14.94882 |
| NOR 16-19 wks 2 A | mmu-miR-125a-5p-4395309 | 20.98321 |
| NOR 16-19 wks 2 A | mmu-miR-125b-5p-4373148 | 18.8161 |
| NOR 16-19 wks 2 A | mmu-miR-126-3p-4395339 | 16.30896 |
| NOR 16-19 wks 2 A | mmu-miR-126-5p-4373269 | 18.47832 |
| NOR 16-19 wks 2 A | mmu-miR-133a-4395357 | 15.29469 |
| NOR 16-19 wks 2 A | mmu-miR-133b-4395358 | 18.64341 |
| NOR 16-19 wks 2 A | mmu-miR-134-4373299 | 20.99735 |
| NOR 16-19 wks 2 A | mmu-miR-139-5p-4395400 | 18.97168 |
| NOR 16-19 wks 2 A | mmu-miR-140-4373374 | 18.91284 |
| NOR 16-19 wks 2 A | mmu-miR-1-4395333 | 19.4555 |
| NOR 16-19 wks 2 A | mmu-miR-145-4395389 | 17.70353 |
| NOR 16-19 wks 2 A | mmu-miR-146a-4373132 | 16.37059 |
| NOR 16-19 wks 2 A | mmu-miR-146b-4373178 | 18.80031 |
| NOR 16-19 wks 2 A | mmu-miR-150-4373127 | 14.81088 |
| NOR 16-19 wks 2 A | mmu-miR-155-4395701 | 21.94143 |
| NOR 16-19 wks 2 A | mmu-miR-16-4373121 | 13.96795 |
| NOR 16-19 wks 2 A | mmu-miR-17-4395419 | 15.05429 |
| NOR 16-19 wks 2 A | mmu-miR-184-4373113 | 23.94063 |
| NOR 16-19 wks 2 A | mmu-miR-186-4395396 | 19.94224 |
| NOR 16-19 wks 2 A | mmu-miR-191-4395410 | 12.27799 |
| NOR 16-19 wks 2 A | mmu-miR-192-4373108 | 16.93415 |
| NOR 16-19 wks 2 A | mmu-miR-193b-4395597 | 18.29602 |
| NOR 16-19 wks 2 A | mmu-miR-195-4373105 | 19.36533 |
| NOR 16-19 wks 2 A | mmu-miR-199a-3p-4395415 | 21.40579 |
| NOR 16-19 wks 2 A | mmu-miR-19a-4373099 | 17.96985 |
| NOR 16-19 wks 2 A | mmu-miR-19b-4373098 | 13.91888 |
| NOR 16-19 wks 2 A | mmu-miR-200b-4395362 | 20.18812 |
| NOR 16-19 wks 2 A | mmu-miR-200c-4395411 | 21.35534 |
| **Sample** | **Detector** | ***C*_T_** |
| NOR 16-19 wks 2 A | mmu-miR-203-4373095 | 19.37577 |
| NOR 16-19 wks 2 A | mmu-miR-204-4373094 | 22.81751 |
| NOR 16-19 wks 2 A | mmu-miR-20a-4373286 | 15.42234 |
| NOR 16-19 wks 2 A | mmu-miR-210-4373089 | 19.84507 |
| NOR 16-19 wks 2 A | mmu-miR-21-4373090 | 16.82838 |
| NOR 16-19 wks 2 A | mmu-miR-214-4395417 | 20.60132 |
| NOR 16-19 wks 2 A | mmu-miR-215-4373316 | 19.33526 |
| NOR 16-19 wks 2 A | mmu-miR-222-4395387 | 14.95893 |
| NOR 16-19 wks 2 A | mmu-miR-223-4395406 | 13.32789 |
| NOR 16-19 wks 2 A | mmu-miR-24-4373072 | 13.74145 |
| NOR 16-19 wks 2 A | mmu-miR-25-4373071 | 19.26147 |
| NOR 16-19 wks 2 A | mmu-miR-27a-4373287 | 21.59117 |
| NOR 16-19 wks 2 A | mmu-miR-296-5p-4373066 | 22.95624 |
| NOR 16-19 wks 2 A | mmu-miR-29a-4395223 | 17.3401 |
| NOR 16-19 wks 2 A | mmu-miR-29c-4395171 | 20.40365 |
| NOR 16-19 wks 2 A | mmu-miR-30a-4373061 | 17.84357 |
| NOR 16-19 wks 2 A | mmu-miR-30b-4373290 | 15.94721 |
| NOR 16-19 wks 2 A | mmu-miR-30c-4373060 | 16.83537 |
| NOR 16-19 wks 2 A | mmu-miR-30d-4373059 | 19.50931 |
| NOR 16-19 wks 2 A | mmu-miR-30e-4395334 | 18.76899 |
| NOR 16-19 wks 2 A | mmu-miR-320-4395388 | 19.37905 |
| NOR 16-19 wks 2 A | mmu-miR-324-3p-4395639 | 21.87233 |
| NOR 16-19 wks 2 A | mmu-miR-328-4373049 | 15.98349 |
| NOR 16-19 wks 2 A | mmu-miR-331-3p-4373046 | 22.19309 |
| NOR 16-19 wks 2 A | mmu-miR-339-3p-4395663 | 20.26835 |
| NOR 16-19 wks 2 A | mmu-miR-342-3p-4395371 | 18.97861 |
| NOR 16-19 wks 2 A | mmu-miR-365-4373194 | 20.9765 |
| NOR 16-19 wks 2 A | mmu-miR-375-4373027 | 18.23378 |
| NOR 16-19 wks 2 A | mmu-miR-409-3p-4395443 | 21.86159 |
| NOR 16-19 wks 2 A | mmu-miR-434-3p-4395734 | 23.00217 |
| NOR 16-19 wks 2 A | mmu-miR-451-4373360 | 17.9532 |
| NOR 16-19 wks 2 A | mmu-miR-484-4381032 | 14.4835 |
| NOR 16-19 wks 2 A | mmu-miR-532-5p-4380928 | 20.70055 |
| NOR 16-19 wks 2 A | mmu-miR-574-3p-4395460 | 16.9939 |
| NOR 16-19 wks 2 A | mmu-miR-672-4395438 | 21.94106 |
| NOR 16-19 wks 2 A | mmu-miR-676-4386776 | 21.9361 |
| NOR 16-19 wks 2 A | mmu-miR-685-4386748 | 18.94612 |
| NOR 16-19 wks 2 A | mmu-miR-744-4395435 | 20.9403 |
| NOR 16-19 wks 2 A | mmu-miR-92a-4373013 | 14.94557 |
| NOR 16-19 wks 2 A | mmu-miR-93-4373302 | 18.34133 |

C_T_ values of miRNA expressed in all samples. These miRNAs were considered as candidates for circulating endogenous control candidates. miRNA names and identification numbers are from the TaqMan® Rodent MicroRNA qRT-PCR array card A v2.0.
